# Electrogenerated Chemiluminescence Coupled with Molecularly Imprinted Polymer for Sensitive and Selective Detection of *N*,*N*-Dimethyltryptamine

**DOI:** 10.1021/acs.analchem.4c06886

**Published:** 2025-03-14

**Authors:** Jesy Alka Motchaalangaram, Paramasivam Mahalingam, Karl J. Wallace, Wujian Miao

**Affiliations:** † Department of Chemistry and Biochemistry, The University of Southern Mississippi, Hattiesburg, Mississippi 39406, United States; ‡ School of Polymer Science and Engineering, The University of Southern Mississippi, Hattiesburg, Mississippi 39406, United States

## Abstract

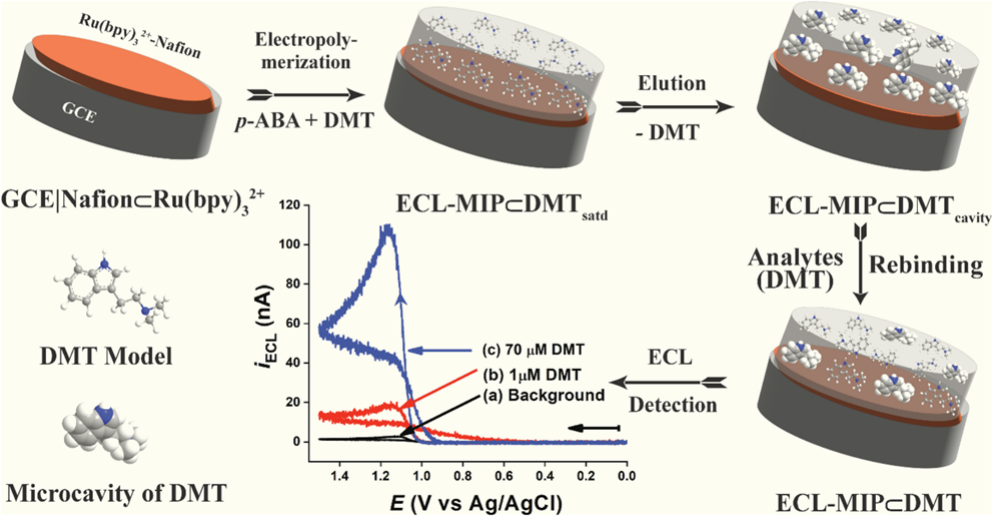

A simple and efficient approach that combined electrogenerated chemiluminescence (ECL) and molecularly imprinted polymers (MIPs) for selective and sensitive detection of the hallucinogenic drug *N,N*-dimethyltryptamine (DMT) was developed. ECL, one of the most sensitive analytical techniques for ultratrace analyte detection, offers the advantage of light-free spectroscopic analysis initiated by electrochemistry. MIPs, on the other hand, provide specific binding sites, allowing the target analyte to become selectively imprinted within the polymer matrix. In this study, an ECL coupled-MIP sensor was fabricated using *para*-aminobenzoic acid (*p*-ABA) as the monomer and DMT as the template molecule. The MIP was electropolymerized onto a glassy carbon electrode coated with a Nafion film entrapping [Ru(bpy)_3_]^2+^ species. Following elution, the imprinted sites were reoccupied by DMT, generating ECL signals in a phosphate buffered solution during anodic potential scanning. The ECL-MIP sensor demonstrated a wide dynamic range for DMT detection, from 0.5 to 300 μM, with an estimated detection limit of 0.5–1.0 μM (*S*/*N* = 3). The sensor’s reproducibility, stability, and selectivity were also evaluated. Finally, density functional theory was employed to investigate the structure–property relationship of the *p*-ABA-DMT interaction. This work demonstrated the potential of ECL coupled with MIP technology for identifying structurally related molecules, achieving enhanced selectivity with a simple and cost-effective design.

## Introduction

The electrogenerated chemiluminescence (ECL) technique offers a broad spectrum of advantages for detecting various analytes, including antigens or antibodies in immunoassays,^1,2^ microRNAs,^3,4^ explosives,^5^ and food allergens,^6^ due to its wide dynamic range, high sensitivity, reproducibility, nondestructive nature, and rapid sample analysis. Among analytical techniques, ECL is particularly promising, as it combines the key characteristics of electrochemistry and photoluminescence. The technique can be precisely controlled through external potential waveforms and scan rates without requiring a light source, which enhances sensitivity and minimizes background signals.^7^ Recent advancements in ECL have enabled the development of point-of-care devices that integrate with smartphones, making ECL a cost-effective, convenient, and user-friendly detection method.^8−10^ When ECL is used to detect analytes with amino functionalities, the amine group can undergo electro-oxidation, forming strong reducing free radicals via deprotonation and generating ECL signals through subsequent energetic chemical oxidation reactions with various ECL emitters (often electro-oxidized) such as ruthenium complexes,^7,11^ cyclometalated iridium complexes,^12^ luminol,^13^ and quantum dots.^14^ However, the redox properties of these amino functionalities frequently occur within similar potential ranges, resulting in overlapping ECL responses. This overlap presents a selectivity challenge, complicating the identification of the ECL signal generated by a specific target analyte. Therefore, it is essential to develop a method that is both highly sensitive and selective—an important criterion for accurately and precisely quantifying target analytes.

The molecular imprinting approach has been employed in this work due to its high selectivity and strong affinity for target analytes through specific binding interactions.^15−17^ Polymers used for molecular imprinting typically rely on electrostatic interactions or a combinational of hydrogen-bonding interactions, which serve as binding domains for specific analytes. Common functional groups in these polymers include electropositive groups such as –NH_2_, –SH, −OH^18−20^ and electronegative moieties like −COOH, −CHO, –NO_2_, −SO_3_H.^21−24^ The specific recognition by molecularly imprinting polymer (MIP) films resembles the selective binding mechanism of antibodies to antigens or probe single-strand DNA to complementary target DNA sequencies.^15^ Furthermore, MIP films are chemically inert and cost-effective.^15^ While methods such as sol–gel processing,^25,26^ RAFT polymerization,^27^ and UV irradiation^28,29^ have been employed, electropolymerization is an especially effective strategy for preparing MIPs. This approach enables control over the morphology and thickness of MIP films by adjusting parameters such as the monomer-to-template ratio, concentrations, scan rate, and the number of cyclic voltammetric cycles used for polymerization.^30,31^ Additionally, electropolymerization enables in situ deposition directly onto the electrode surface, an optimal strategy for on-site analyte detection that avoids complex synthetic procedures. Therefore, coupling MIPs with the ECL strategy provides a quantitative analytical platform that addresses sensitivity and selectivity for analyte detection.

**Figure 1:**
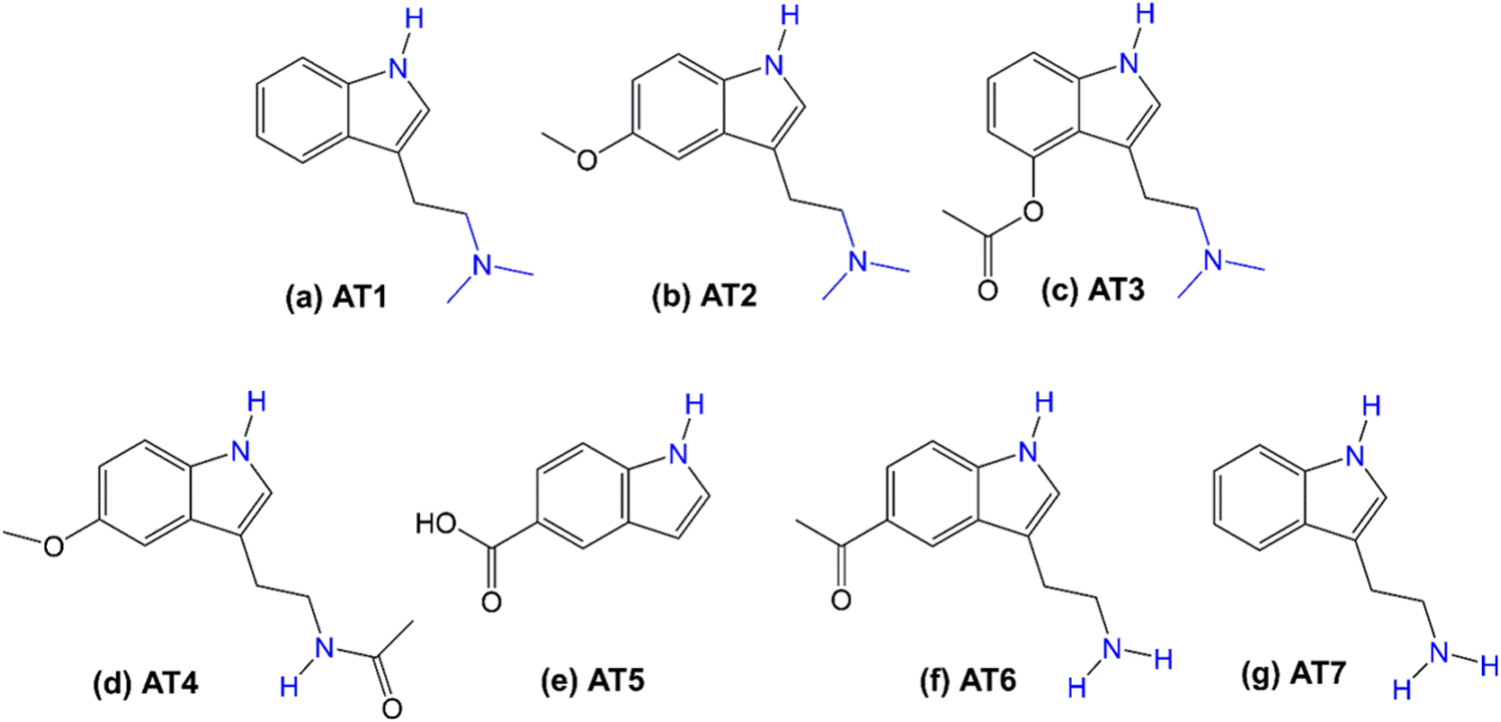
Molecular structures of various analytes (AT). (a) AT1: *N,N*-dimethyltryptamine (DMT), (b) AT2: 5-methoxy-*N*,*N*-dimethyltryptamine, (c) AT3: 4-acetoxy-*N*, *N*-dimethyltryptamine, (d) AT4: melatonin, (e) AT5: indole-5-carboxylic acid, (f) AT6: 5-acetyl tryptamine, and (g) AT7: tryptamine.

In this study we have used a *para*-aminobenzoic acid (*p*-ABA) monomer to prepare MIP films, as both the –NH_2_ and −COOH groups are excellent hydrogen bond donor–acceptors to bind analytes via hydrogen bonding and electrostatic interactions. The goal of this research is to develop an effective technique for detecting illicit drugs containing amino functionalities. For this purpose, *N*,*N*-dimethyltryptamine (DMT, Figure 1a), a hallucinogenic tryptamine alkaloid found in many plants,^32−34^ was chosen as the template. The molecular structure of DMT shares similarities with human neurotransmitters, specifically serotonin and melatonin.^35^*N*,*N*-Dimethyltryptamine is often called the “spirit molecule” due to its ability to induce profound hallucinations within 90–120 s when consumed orally at doses above 0.2 mg/kg.^36^ Existing techniques for DMT detection, such as gas chromatography–mass spectrometry (GC-MS),^37^ liquid chromatography–mass spectrometry (LC-MS),^38,39^ and high performance liquid chromatography–mass spectrometry (HPLC-MS),^40^ are limited by high instrumentation costs, lack of portability, frequent need for derivatization, and requirements for highly skilled operators.

In this study, [Ru(bpy)_3_]^2+^, a luminophore with fully reversible redox characteristics, was used as an efficient ECL emitter.^7^*p*-ABA monomer and DMT were electropolymerized onto a glassy carbon electrode (GCE) coated with a Nafion film containing [Ru(bpy)_3_]^2+^. The resulting MIP film was formed on the electrode surface, providing mechanical stability in aqueous solutions, with no leaching or cracking observed.^41−43^ The selectivity of the MIP film was evaluated against a series of six DMT derivatives (Figure 1b–g), showing that DMT exhibited approximately six times higher selectivity over the DMT derivatives. To qualitatively assess the structure–property relationship of the *p*-ABA-DMT interaction, we employed density functional theory (DFT) methods. DFT studies showed that the preferable outward orientation of carboxylic acid groups forms an asymmetric electronegative region, which serves as an effective binding site for DMT.

## Experimental Section

### Reagents

All chemicals were used as received, with details provided in the Supporting Information. A 0.10 M phosphate buffer solution (PBS, pH 7.4) was used as the working solution, and analytes were dissolved in PBS after MeOH evaporation under N_2_ gas to prevent electrochemical oxidation.

### Electrochemical and ECL Setup

Electrochemical measurements were performed using a CH Instruments Model 660A workstation with a three-electrode configuration: a glassy carbon working electrode (GCE), an Ag/AgCl reference electrode, and a platinum counter electrode. ECL signals were captured using a Hamamatsu R928 photomultiplier tube integrated into a custom CV-ECL setup.

### FTIR Analysis

Fourier Transform Infrared Spectroscopy (FTIR) spectra were obtained to confirm functional groups using a Thermo Fisher Everest Nicolet Summit diamond crystal ATR-FTIR spectrometer. MIP⊂DMT films were electrodeposited onto FTO glass from a PBS solution containing 4.0 mM *p*-ABA and 1.0 mM DMT.

### ECL-MIP⊂DMT Sensor Fabrication

**Scheme 1:**
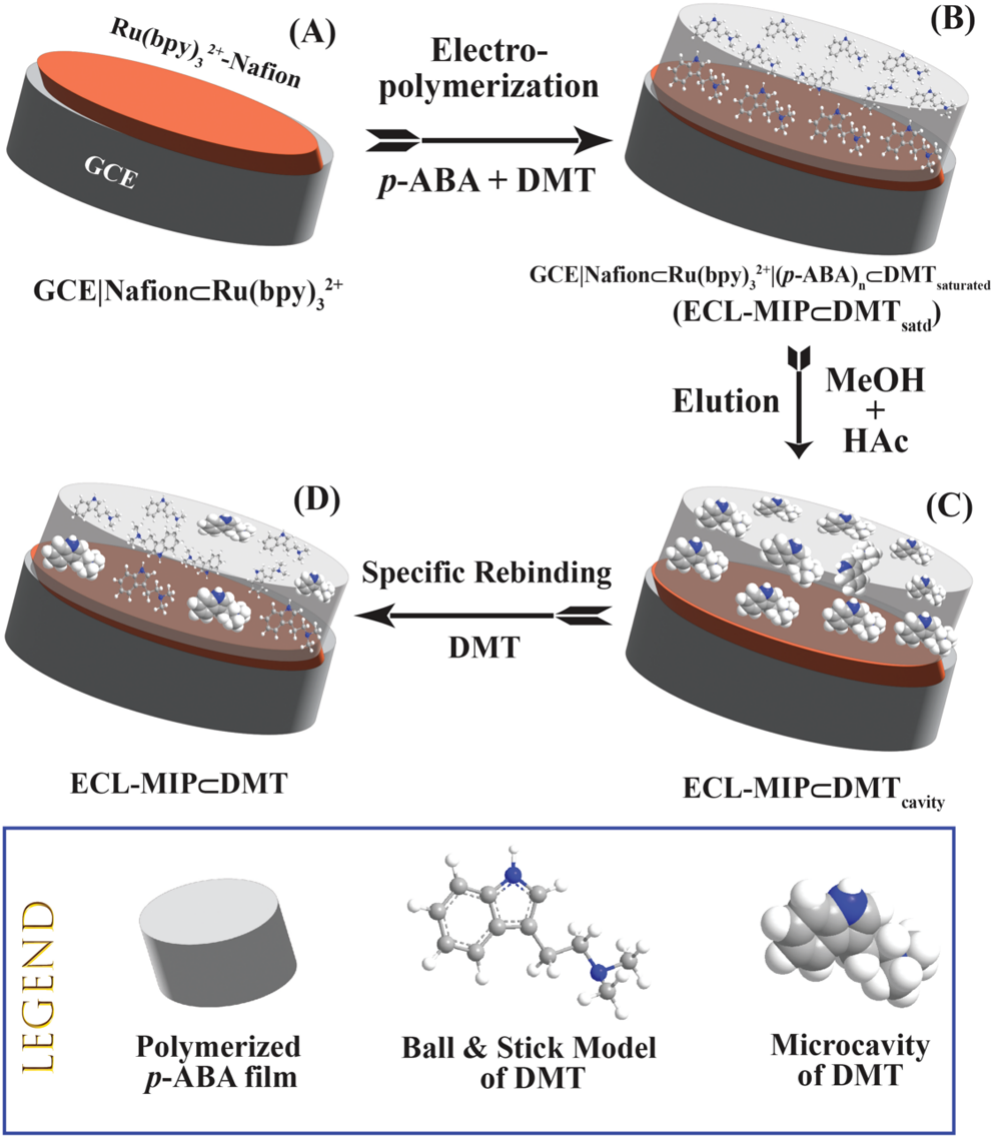
Fabrication Processes of Electrogenerated Chemiluminescence (ECL) Coupled with Molecularly Imprinted polymer (MIP) Sensor for Sensitive and Selective Detection of DMT (A) A GCE casted with a Nafion film containing [Ru(bpy)3]^2+^ (GCE|Nafion⊂[Ru(bpy)^3^]^2+^), (B) A DMT-imprinted *p*-ABA polymer film on the GEC from step (A) (ECL-MIP⊂DMTsatd), (C) Creation of DMT-specific nanocavities after elution (ECL-MIP⊂DMTcavity), and (D) The final ECL-MIP⊂DMT sensor with some nanocavities rebounding the target analyte DMT.

Scheme 1 illustrates the fabrication of the ECL-MIP⊂DMT sensor. A GCE is modified with a Nafion⊂[Ru(bpy)_3_]^2+^ film to form the GCE|Nafion⊂[Ru(bpy)_3_]^2+^ electrode, where ‘|’ denotes the interface between the GCE and the Nafion film, and ‘⊂’ indicates the inclusion of [Ru(bpy)_3_]^2+^ within the Nafion matrix (Scheme 1A). The electrode is then subjected to electropolymerization of *p*-ABA with DMT to create MIP films. The fully loaded-DMT electrode is designated as GCE|Nafion⊂[Ru(bpy)_3_]^2+^|(*p*-ABA)_n_⊂DMT_saturated_, simplified as ECL-MIP⊂DMT_satd_ (Scheme 1B). To generate nanocavities for specific DMT binding, the DMT was removed from the MIP matrix using a MeOH/HAc solution, resulting in the ECL-MIP⊂DMT_cavity_ electrode (Scheme 1C). ECL detection is subsequently performed with the ECL-MIP⊂DMT electrode (Scheme 1D), where the target analyte DMT binds selectively to the nanocavities within the MIP film.

## Results and Discussion

### Fourier Transform Infrared Spectroscopy (FTIR)

The carboxylic FTIR analysis confirmed successful electropolymerization of *p*-ABA. The monomer spectrum (Figure S1a in Supporting Information) showed the NH_2_ stretching vibrations at ∼3459 and ∼3360 cm^–1^, indicating “free-state” amino groups, while carboxylic groups exhibited hydrogen bonding, evidenced by a broad O–H region (∼3000–2800 cm^–1^) and a C=O stretch at ∼1658 cm^–1^.^44^ Benzene C=C stretches (∼1590–1540 cm^–1^) and C–N vibrations (∼1285 cm^–1^) were also observed. In the polymer spectrum (Figure S1b in Supporting Information), a broad band at ∼3215 cm^–1^ indicated the conversion of primary to secondary amines during electropolymerization (see the following section for further explanation).^45−47^

### Electrochemical Characterization of GCE|Nafion⊂[Ru(bpy)_3_]^2+^ Electrode

The GCE|Nafion⊂[Ru(bpy)_3_]^2+^ electrode was characterized using CV technique in 0.10 M PBS (pH 7.4) at varying scan rates. At 10 mV/s, the trapped [Ru(bpy)_3_]^2+^ complex within the Nafion film exhibited a typical solution-phase reversible redox reaction, with anodic and cathodic peak potentials observed at ∼ 1.11 V and ∼ 1.04 V vs Ag/AgCl, respectively (Figure S2 in SI). Increasing the scan rate (10–150 mV/s) caused peak separation (Δ*E*_p_) to widen from ∼70 to ∼120 mV due to the iR drop. Peak currents showed a linear relationship with the square root of scan rate (Insert in Figure S2), confirming that the [Ru(bpy)_3_]^2+^ ions confined within the Nafion film follow a diffusion-controlled redox process.^48−50^

### Electropolymerization of *p*-ABA

**Figure 2:**
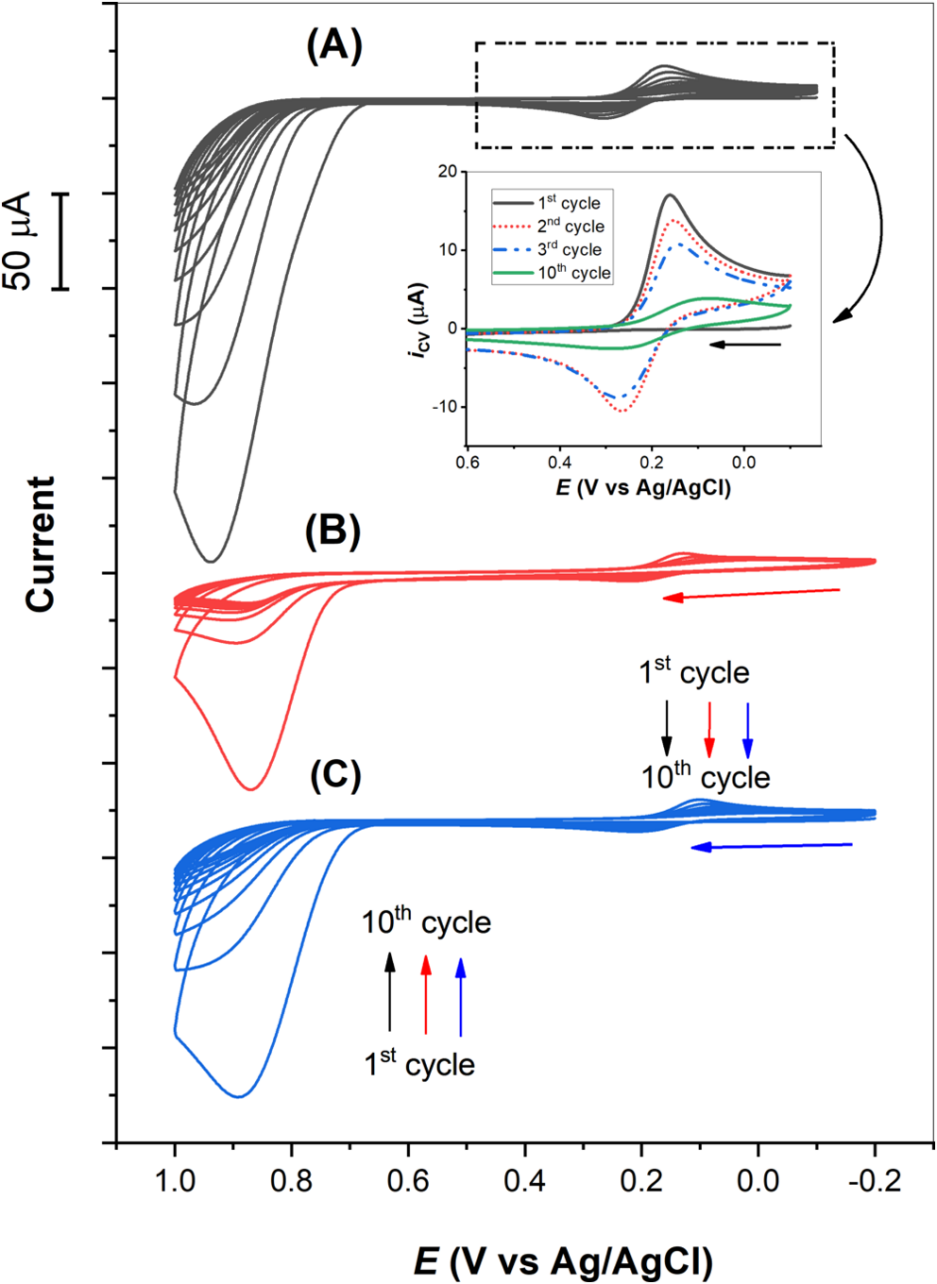
First ten CV cycles of electropolymerization of 4.0 mM *p*-ABA in 0.10 M PBS (pH 7.4) on an electrode of (A) GCE, (B) GCE|Nafion, and (C) GCE|Nafion⊂[Ru(bpy)_3_]^2+^ (with 1.0 mM DMT template in solution), respectively, at a scan rate of 50 mV/s.

**Scheme 2:**
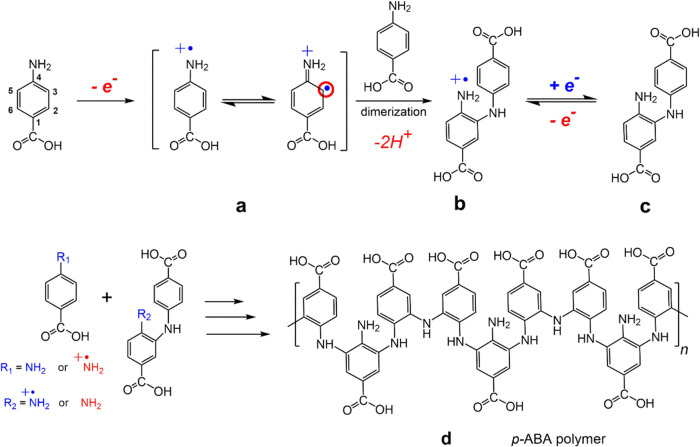
Proposed Eelectropolymerization Scheme for the Preparation of *p*-ABA polymer (a) Electrochemical oxidation of *p*-ABA monomer, (b) Dimer formation via subsequent chemical reactions, (c) Quasi-reversible redox reactions of the formed dimer, (d) Propagation reactions leading to polymer chain growth

Figure 2 presents the initial 10 CV cycles of electropolymerization of a 4.0 mM *p*-ABA in 0.10 M PBS (pH 7.4) on an electrode of (A) GCE, (B) GCE|Nafion, and (C) GCE|Nafion⊂[Ru(bpy)_3_]^2+^ (with 1.0 mM DMT template in solution) at a scan rate of 50 mV/s, respectively. To maximize *p*-ABA polymerization while minimizing [Ru(bpy)_3_]^2+^ oxidation in Nafion during the fabrication of ECL-MIP sensors, the anodic potential was stopped at 1.0 V vs Ag/AgCl for all studies. In Figure 2A, a large irreversible oxidation peak appears at 0.89 V vs Ag/AgCl during the first scan. This indicates the formation of amino cation radicals upon *p*-ABA oxidation (Scheme 2). The nature of substituents on the aromatic ring typically dictates the activation site for electrophilic substitution via both resonance and inductive effects. The amino group in the aromatic ring acts as a strong activating group, directing further substitution to the *ortho-para* positions, while the carboxylic acid group, is a moderately deactivating group, which directs additional substitution to the *meta* position. In the case of *p*-ABA, the lone pair electrons on the nitrogen atom of the amino group promotes π-delocalization within the aromatic ring, strongly activating the *ortho-para* positions for electrophilic radical substitution; however, as the *para* position is blocked by the acid group, *p*-ABA. electropolymerization will only occur at the 3 and 5 positions (Scheme 2a). Consequently, a second amino radical cation favors polymerization through the *ortho* position, forming a dimer product with the elimination of two hydrogen atoms (Scheme 2b).^42,51^ The resulting dimer exhibits quasi-reversible redox behavior with reduction and oxidation peaks at ∼0.12 V and ∼0.21 V vs Ag/AgCl, respectively (Scheme 2c and the inset in Figure 2).^51^ This coupling reaction will continue to occur at the 3 or 5 positions, but steric hindrance at the *ortho* positions during polymerization inhibits coupling at position 5 (see DFT section). With successive potential cycles, the electrode current gradually decreases, and the oxidation and reduction potentials shift positively and negatively, respectively. This shift is attributed to the increased kinetic resistance of the polymer film and the slower diffusion of oligomeric species compared to monomers. Notably, CV responses (Figure 2B vs C) did not significantly differ with or without DMT present, likely because DMT oxidation occurs at ∼0.8 V vs Ag/AgCl, which partially overlaps with the initial oxidation of *p*-ABA, and the concentration of DMT (1.0 mM) is much lower than that of *p*-ABA (4.0 mM). When electropolymerization of *p*-ABA is conducted in the presence of DMT, a MIP is formed. Otherwise, the resulting polymer film is a nonimprinted polymer (NIP, Scheme 2d). Figure S3 in Supporting Information shows an AFM image and the corresponding height profile of an NIP modified FTO electrode.

### Electrochemical Characterization of Modified Films

**Figure 3:**
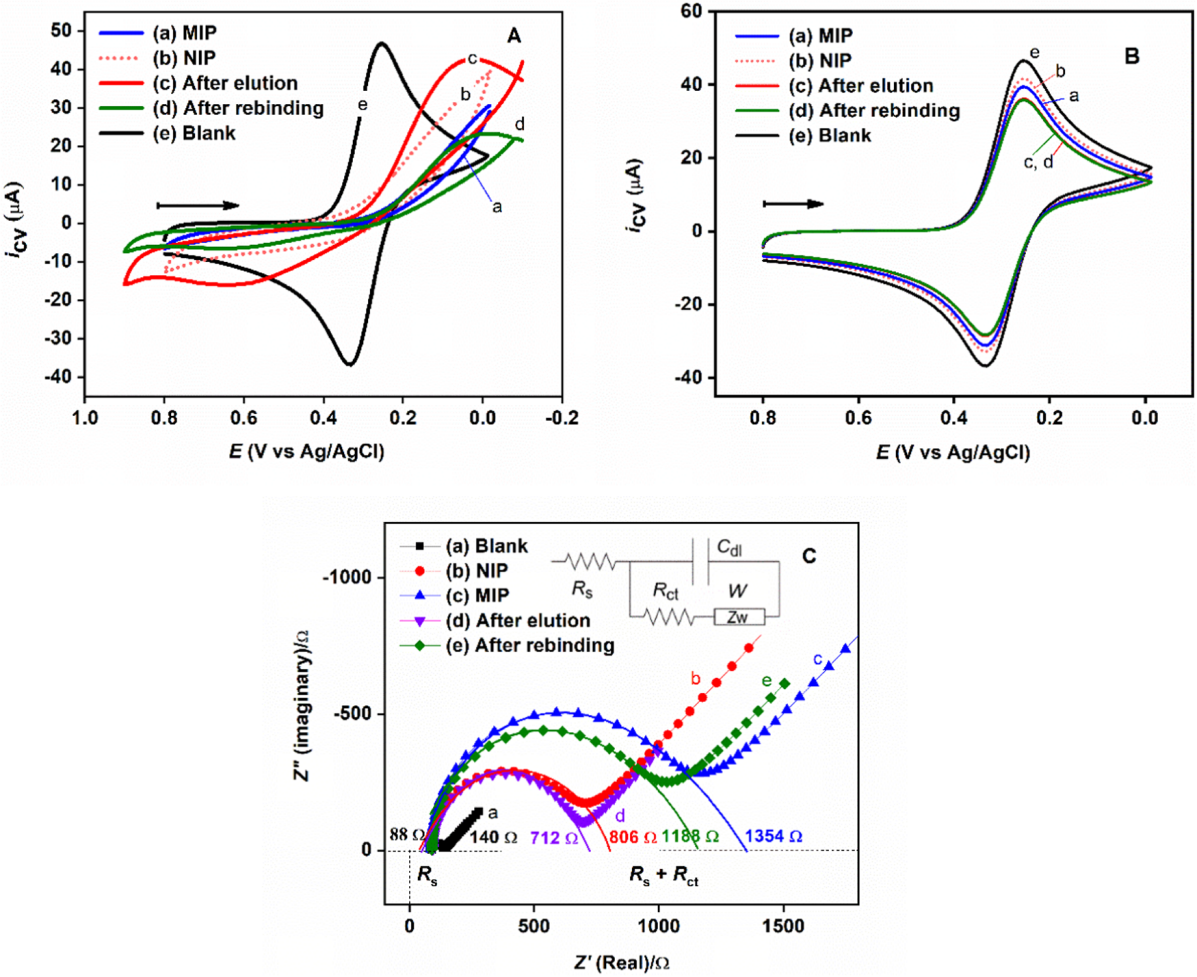
(A) Cyclic voltammograms of modified electrodes in 5.0 mM K_3_[Fe(CN)_6_] with 0.10 M PBS (pH 7.4) at 50 mV/s. (B) Cyclic voltammograms in 5.0 mM ferrocene methanol with 0.10 M PBS (pH 7.4) at 50 mV/s. (C) Nyquist plots of modified electrodes in 5.0 mM K_3_Fe(CN)_6_ – 5.0 mM K_4_Fe(CN)_6_ mixture with 0.10 M PBS (pH 7.4). Insert: Equivalent circuit used to fit EIS data for estimating *R*_s_ and *R*_ct_ values, with *C*_dl_ and *W* representing double-layer capacitance and Warburg impedance, respectively.

The modified electrodes were characterized using CV in 5.0 mM K_3_[Fe(CN)_6_] solution prepared in 0.10 M PBS (pH 7.4). As shown in Figure 3Aa (blue line), no significant CV response is observed at a freshly prepared MIP electrode (i.e., GCE|Nafion⊂[Ru(bpy)_3_]^2+^|(*p*-ABA)*_n_*⊂DMT_saturated_). This lack of response is likely due to strong repulsive electrostatic interactions between the negatively charged [Fe(CN)_6_]^3–^ and the negatively charged (*p*-ABA)*_n_*⊂DMT_saturated_ electrode surface, as the majority of the carboxylic groups in the *p*-ABA polymer film are expected to be deprotonated (*p*-ABA has a p*K*_a_ of ∼4.8) under the experimental conditions. Different electrodes are anticipated to exhibit distinct surface morphologies and charges, which affect the redox behavior of [Fe(CN)_6_]^3–^. This may explain why the CV current at the NIP (Figure 3Ab, orange dotted line) and MIP after DMT elution (Figure 3Ac, red line) and rebinding (Figure 3Ad, green line) is lower than that observed at the bare GCE (Figure 3Ae, black line). As expected, when the negatively charged [Fe(CN)_6_]^3–^ ions are replaced with neutral ferrocene methanol molecules, no dramatic changes in CV responses are observed for the above electrodes (Figure 3B).

Electrochemical impedance spectroscopy (EIS) was used to investigate the interfacial properties of the modified electrode surface. The EIS measurements were conducted in a solution containing 5.0 mM K_3_Fe(CN)_6_ – 5.0 mM K_4_Fe(CN)_6_ mixture with 0.10 M PBS (pH 7.4) as the supporting electrolyte at the formal redox potential of 0.30 V vs Ag/AgCl with an alternating voltage of 5 mV. As shown in Figure 3C, the bare and all modified electrodes show a nearly identical solution resistance (*R*_s_) of ∼88 Ω, as the same redox couple [Fe(CN)_6_]^3–^/[Fe(CN)_6_]^4–^ was used. However, an increase in charge transfer resistance (*R*_ct_) is observed, as indicated by the larger semicircle diameter in the Nyquist plot, which was analyzed using an equivalent circuit model (Insert in Figure 3C). Due to the electrostatic repulsion effect, the *R*_ct_ value rises from ∼ 140 Ω for the bare GCE to ∼806 Ω for the NIP, and further to ∼1354 Ω for the MIP electrode (Figure 3Ca–c). After elution, the *R*_ct_ value significantly decreases to ∼712 Ω, (Figure 3Cd), indicating enhanced diffusion of the [Fe(CN)_6_]^3–^/[Fe(CN)_6_]^4–^ species. Upon DMT rebinding to the MIP electrode, the *R*_ct_ value increases again to ∼1188 Ω (Figure 3Ce), suggesting successful DMT molecule uptake. These results are consistent with the CV data and demonstrate the successful fabrication of the ECL-MIP sensor.

### Optimization of ECL-MIP Sensor Operating Conditions

Several parameters influence the performance of the ECL-MIP sensors,^42,52^ including the concentration and ratio of monomer to template, the number of electropolymerization cycles, and the elution and rebinding time. These factors impact the chemical, physical, or mechanical stability and reproducibility of the electropolymerized film, as well as the integrity of the inner Nafion⊂[Ru(bpy)_3_]^2+^ layer on the GCE. All optimization studies were conducted using ECL under the same experimental settings described above.

#### Effect of Monomer Concentration

**Figure 4:**
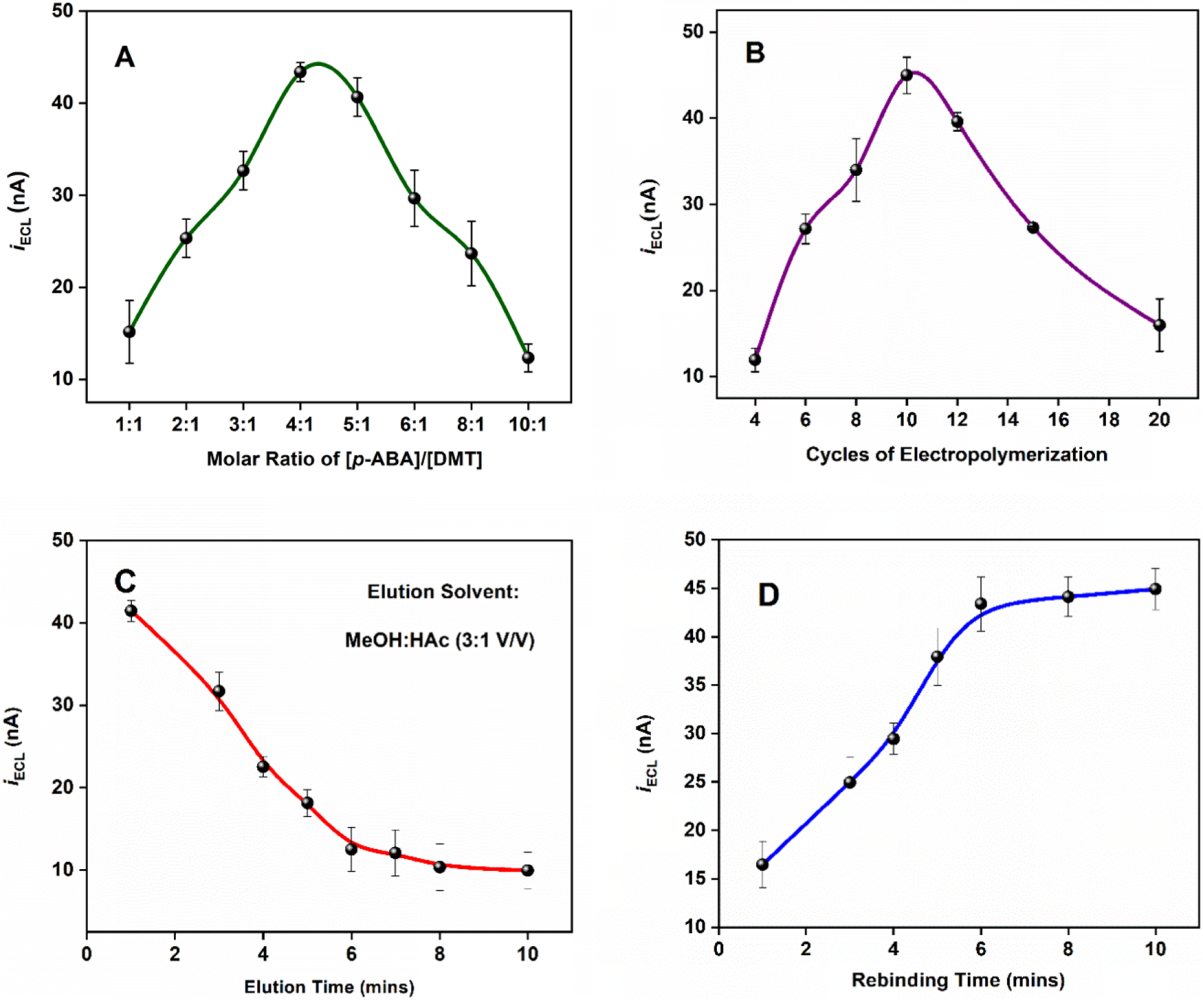
Effect of various experimental conditions on the performance of the DMT-specific ECL-MIP sensor: (A) Molar ratio of *p*-ABA monomer concentration [*p*-ABA] to DMT template concentration [DMT], with a constant [DMT] = 1.0 mM, (B) Number of electropolymerization cycles between −0.20 and 1.00 V vs Ag/AgCl at a scan rate of 50 mV/s, (C) Elution time using MeOH:HAc (3:1 v/v), and (D) Rebinding time with ECL measured using 5.0 μM DMT in 0.10 M PBS (pH 7.4).

At a fixed concentration of 1.0 mM DMT, the impact of monomer concentration on electropolymerization was studied. The thickness of the film and formation of specific binding sites, both crucial to the ECL-MIP sensor’s performance, are dependent on the monomer concentration. The ECL intensity is found to be maximized at 4.0 mM *p*-ABA, as shown in Figure 4A, indicating optimal performance when the molar ratio is 4:1 *p*-ABA/DMT, respectively.

#### Effect of Potential Cycles during Electropolymerization

The number of electropolymerization cycles affects the polymer film thickness, which in turn influences the sensor’s sensitivity and selectivity. A thin film limits DMT bonding capacity, resulting in a weak ECL signal, while a thick film hinders the diffusion of DMT cations and free radicals to reach the [Ru(bpy)_3_]^2+/3+^ species within the Nafion film, reducing ECL production. Using a 4.0 mM *p*-ABA and 1.0 mM DMT solution, MIP generated with 10 CV cycles between −0.20 and 1.00 V vs Ag/AgCl demonstrates the highest ECL response (Figure 4B). Beyond 10 cycles, the ECL signal decreases due to excessive film thickness. Moreover, at very slow scan rates, the resulting film was smooth and dense, making it difficult to remove the template from the film. Therefore, 10 electropolymerization cycles at a scan rate of 50 mV/s were chosen as the optimal conditions.

#### Effect of Elution and Rebinding Time

A suitable solvent is essential for effectively removing the prebound template from the polymer network without compromising the MIP framework. Additionally, template-specific binding sites and their functionalities must remain intact to enable recognition during the rebinding process. DMT, a relatively small organic base, is highly soluble in methanol and acetic acid. Within the MIP, DMT predominantly exists in the protonated form (DMT-H^+^) in pH 7.4 PBS buffer, given its p*K*_a_ of ∼ 8.7.^53^ Thus, a methanol and acetic acid (both 99+%) mixture (3:1, v/v) was selected as the eluent. The acidic medium provides H^+^ ions that compete with the DMT-H^+^ template engaged in bonding with the polymer.^54−56^ As shown in Figure 4C, ECL intensity decreases rapidly during the first 6 min of elution, then declines gradually. Considering the potential but minimal leakage of [Ru(bpy)_3_]^2+^ from Nafion in the MeOH-HAc mixture over time, 6 min was determined to be the optimal elution time. Rebinding studies, shown in Figure 4D, were conducted with 5.0 μM DMT in 0.10 M PBS (pH 7.4). This is because, unlike MeOH:HAc, which disrupts interactions between DMT and the imprinted polymers, PBS not only maintains a stable and suitable pH environment for the selective and effective rebinding of DMT to the imprinted sites but also provides an optimal reaction medium for subsequent ECL detection. The ECL intensity initially increases almost linearly, reaching equilibrium at around 6 min, indicating optimal rebinding conditions.

### ECL Detection of DMT

**Figure 5:**
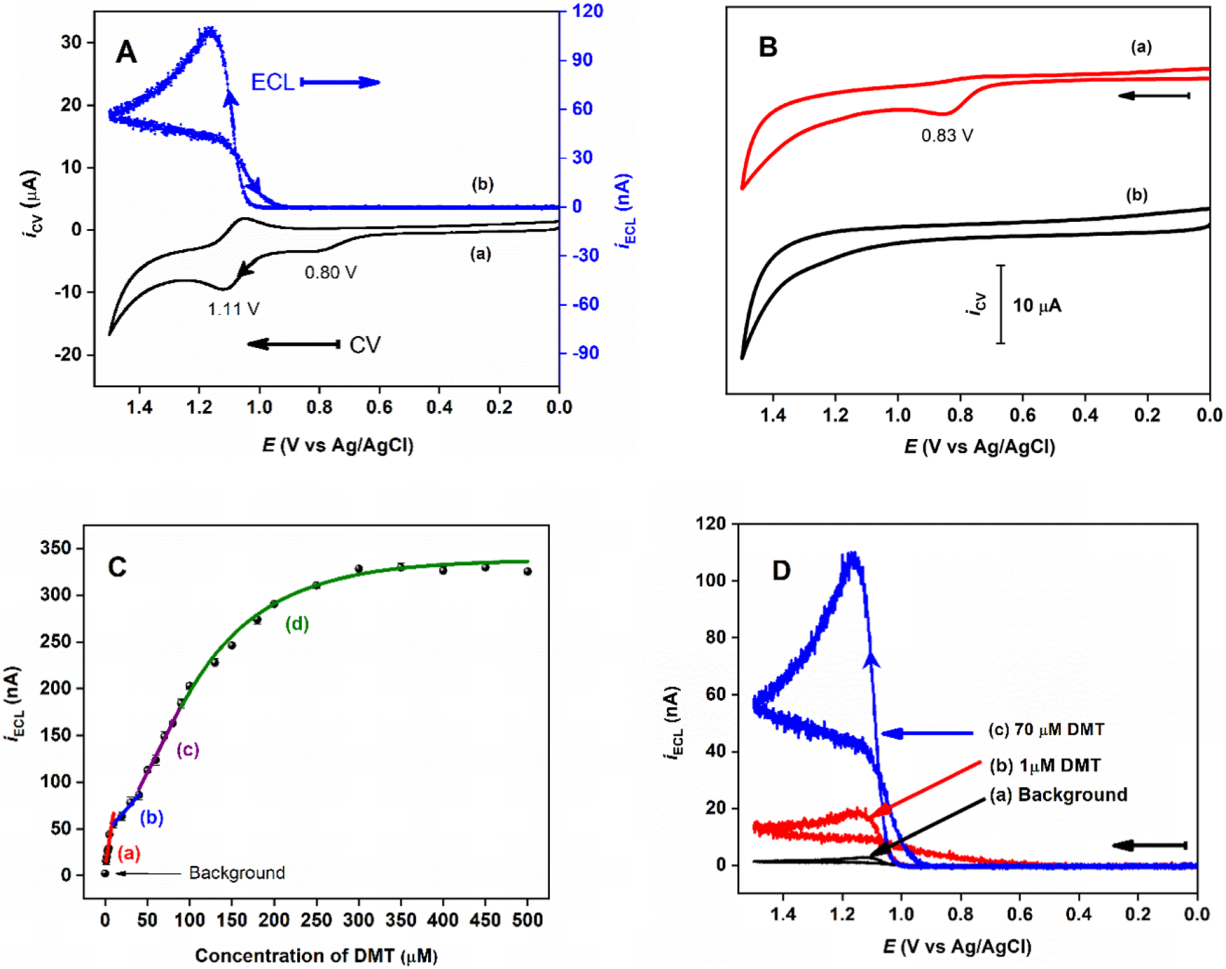
(A) (a) CV and (b) ECL responses of a MIP electrode incubated with 70 μM DMT (e.g., GCE|Nafion⊂[Ru(bpy)_3_]^2+^|(*p*-ABA)*_n_*⊂DMT) in 0.10 M PBS buffer (pH 7.4) at a scan rate of 50 mV/s. (B) CVs of 0.10 M PBS buffer (pH 7.4) (a) with and (b) without 70 μM DMT, using a 3 mm GCE at a scan rate of 50 mV/s. (C) ECL intensity as a function of DMT concentration, showing three linear ranges of (a–c) and a nonlinear range of (d). (D) ECL responses for varying DMT concentrations: (a) 0, (b) 1.0, and (c) 70.0 μM.

**Scheme 3:**
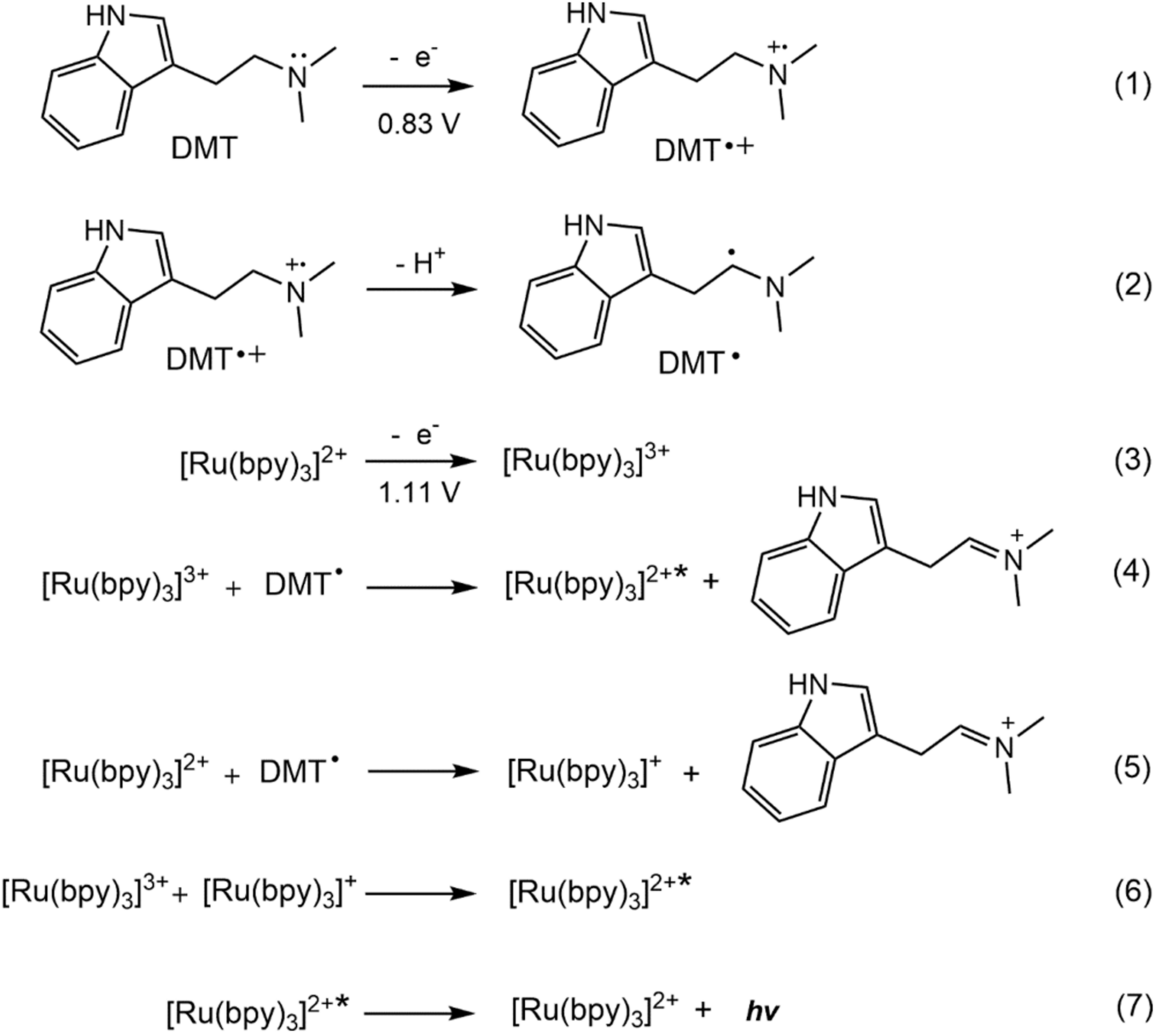
Proposed Anodic ECL Mechanism of the GCE|Nafion⊂[Ru(bpy)_3_]^2+^|(*p*-ABA)_n_⊂DMT Sensor in PBS Buffer (pH 7.4)

Figures 5Aa,b present the CV and ECL responses of the GCE|Nafion⊂[Ru(bpy)_3_]^2+^|(*p*-ABA)*_n_*⊂DMT electrode. During the anodic scan, both the DMT molecules adsorbed on the electrode surface and the immobilized [Ru(bpy)_3_]^2+^ within the Nafion film are oxidized successively, generating DMT**˙**^**+**^ (eq 1 in Scheme 3) and [Ru(bpy)_3_]^3+^ (eq 3), respectively. As illustrated in Figure 5Ba, DMT in solution shows an irreversible oxidation peak at ∼ 0.83 V vs Ag/AgCl at a GCE, which aligns with the observed oxidation wave at ∼ 0.80 V vs Ag/AgCl in Figure 5Aa. This oxidation process likely results from electron removal from the lone pair on the tertiary amine group (eq 1). The resulting DMT**˙**^**+**^ cation radical then undergoes deprotonation, forming a strong reducing DMT**˙** intermediate (eq 2). This intermediate reacts with electrogenerated [Ru(bpy)_3_]^3+^, producing an excited-state [Ru(bpy)_3_]^2+^* (eq 4), which emits surface-confined ECL (eq 7). Alternatively, the DMT**˙** free radical may react with the immobilized [Ru(bpy)_3_]^2+^ to form [Ru(bpy)_3_]^+^ (eq 5). This species can further react with newly produced [Ru(bpy)_3_]^3+^, resulting in an ion annihilation pathway for the formation of excited-state [Ru(bpy)_3_]^2+^* (eq 6). Scheme 3 presents the proposed anodic ECL mechanism of the ECL-MIP|DMT sensor in PBS buffer (pH 7.4).^7,11,57^

### Calibration Curve and Limit of Detection

Once the optimized experimental conditions had been established, the effect of DMT concentration on the ECL intensity of the ECL-MIP sensor was studied. As shown in Figure 5C, the ECL intensity increases with the rising [DMT] across three specific linear regions (Figure 5Ca–c and Table 1) before gradually leveling off (Figure 5Cd). This behavior differs from that observed in typical ECL-based chemical or biochemical sensors, where ECL intensity changes linearly with log[Analyte].^58,59^ This unusual result suggests that DMT binding to the MIP follows a monolayer “adsorption” Langmuir isotherm at very low concentrations and transitions to multilayer “adsorption” extended Langmuir isotherms (or the EBT isotherm model) at higher concentrations.^59,60^ In other words, the data in Figure 5C indicate that binding between DMT analyte and the MIP occurs within a three-dimensional, multilayer porous polymer framework. Similar “atypical” behavior has been reported previously for nitrogen adsorption isotherms in microporous or mesoporous network polymers.^61,62^ The calibration curve (Figure 5C) with a wide dynamic range of 0.5 to 300 μM DMT displays characteristics of a type II isotherm,^63^ which is consistent with the ordered 3D molecular porous structure of the MIP polymer film described in this paper. Notably, as shown in Figure 5Cd, the MIP electrode reaches saturation at [DMT] ≥ ∼300 μM, corresponding to an ECL response of ∼328 nA. Thus, the ECL response from a freshly prepared MIP electrode (i.e., GCE|Nafion⊂[Ru(bpy)_3_]^2+^|(*p*-ABA)*_n_*⊂DMT_saturated_) is approximately 328 nA.

**Table 1:** Least Squares Fitting of Calibration Curve in Figure 5C

linear region	[DMT] (μM)	fitting equation	Pearson’s *r*
a	1.0–10.0	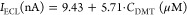 8	0.933
b	10.0–40.0	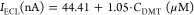 9	0.992
c	40.0–90.0	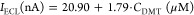 10	0.996

The limit of detection (LOD) for DMT was estimated to be 0.5–1.0 μM (*S*/*N* = 3). This detection limit is notably higher than the nM-range LOD reported for tri-*n*-propylamine (TPrA) in the [Ru(bpy)_3_]^2+^/TPrA system when using techniques such as capillary electrophoresis^7^ or covalent coupling of [Ru(bpy)_3_]^2+^ at an electrode.^60^ However, it is only slightly higher than the ∼ 0.1 μM LOD observed for TPrA in the solution-phase [Ru(bpy)_3_]^2+^/TPrA system.^64^ Several factors may contribute to this difference. First, the structural differences between DMT and TPrA affect the stability and reduction potentials of their respective intermediate radicals (DMT^•^ and TPrA^•^). Second, electrode surface modifications may limit the diffusion and interaction of the ECL emitter [Ru(bpy)_3_]^2+^ with the analyte DMT within the Nafion and MIP films. While the current system provides excellent selectivity, these factors lead to a decrease in ECL intensity, ultimately resulting in a relatively higher detection limit. Figure 5D compares the ECL responses for (a) blank, (b) 1.0 μM DMT, and (c) 70.0 μM DMT, respectively. Table 2 lists several detection methods, along with their linear ranges, and limits of detection. The ECL-MIP sensor presented here offers the advantage of ease of use and cost-effectiveness over conventional chromatography–mass spectrometry analytical techniques, while providing a competitive dynamic range and detection limit.

**Table 2:** Comparison of Different Techniques for the Detection of DMT

method	linear range/μM	LOD/μM	ref
HPLC/ESI-MS		0.00053	(40)
HPLC/Electrospray MS	0.26–5.3	0.053	(65)
LC/MS	0.03–26.6	0.016	(38)
LC/MS		0.53	(39)
GC/MS	8.29–1590	4.14	(37)
GCE/BMIMNTF_2_/ZnTRP	0–3.5	1.75	(66)
ECL-MIP	1.0–90 (3 regions)	0.5–1.0	this study

### Selectivity, Reproducibility, and Stability

#### Selectivity

**Figure 6:**
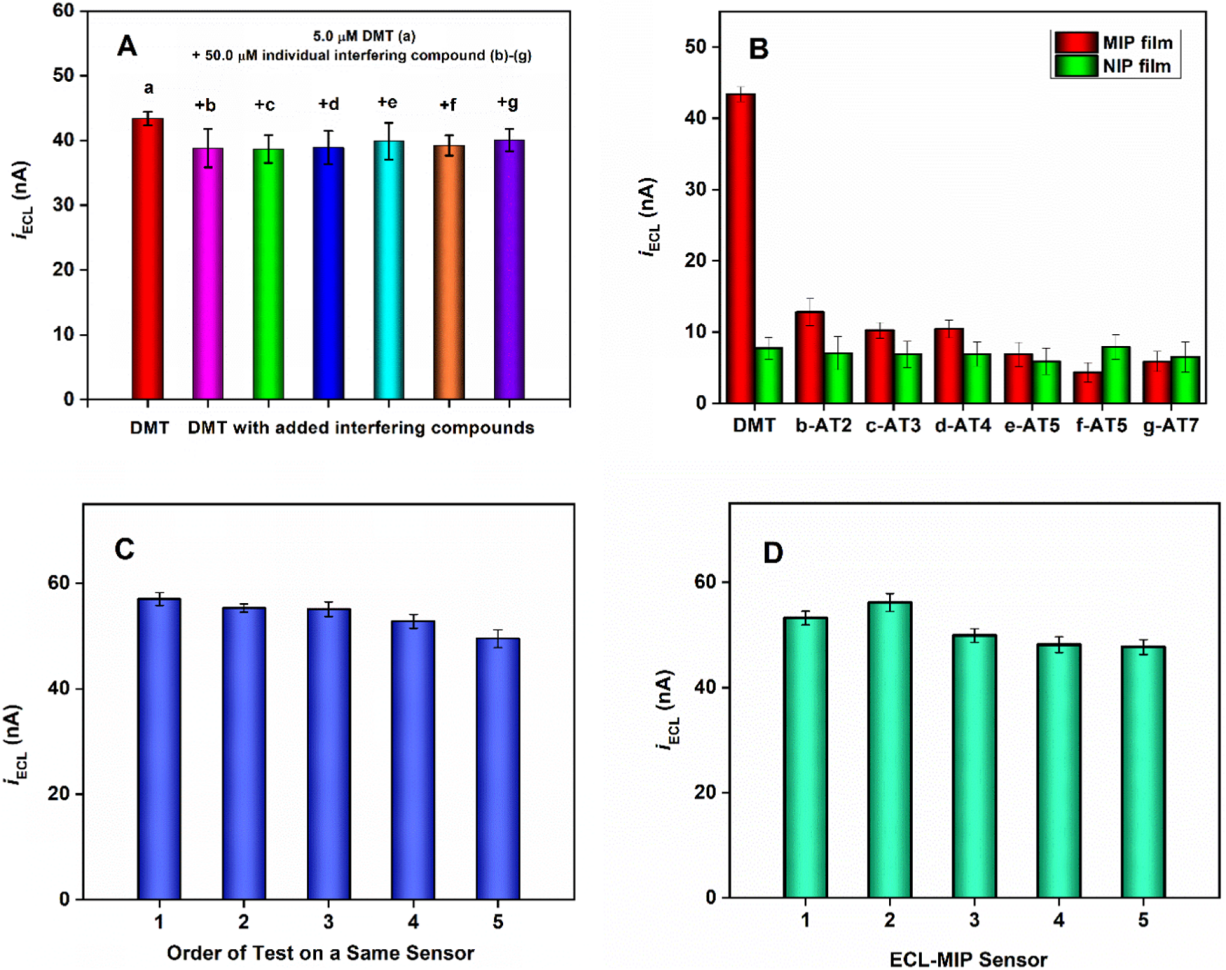
(A) Selective tests of the ECL-MIP sensor with DMT and its six structural analogues. (B) Comparison of ECL signals between the ECL-MIP sensor and the ECL-NIP electrode for DMT and its six analogues, each tested at a concentration of 5.0 μM. (C) Reproducibility studies for the same ECL-MIP sensor over multiple runs with 8.0 μM DMT. (D) Reproducibility studies of the ECL-MIP sensor across five different electrodes with 8.0 μM DMT.

In this study, structurally similar interfering compounds (Figure 1b–g) were selected to evaluate the selectivity of the ECL-MIP sensor. The ECL intensity remains nearly unchanged, after adding 50.0 μM each interfering compound into 5.0 μM DMT solution (Figure 6A), demonstrating that a 10-fold increase in interfering analytes has almost no effect on DMT detection. Additionally, selectivity can be assessed by calculating the imprinting factor (IF, eq 11),^30,42^ where a high IF value indicates strong selectivity
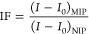
11where *I*_0_ is the blank ECL signal and *I* is the ECL signal in the presence of the target analyte (i.e., DMT) or other interferents. The IF factor for DMT is 6.6, indicating that the net ECL signal from the ECL-MIP sensor is approximately 6.6 times higher than that from the NIP-ECL sensor, as shown in Figure 6B. Other interferents show significantly lower affinities for the ECL-MIP sensor, with typical IF values around 1.2.

#### Reproducibility

The reproducibility of the sensor was tested by monitoring ECL signal changes using an 8.0 μM DMT standard solution. As shown in Figure 6C, repeated measurements on the same sensor results in a relative standard deviation (RSD) of 3.8% across five measurements. Similarly, measurements taken with five different sensors show an RSD of 3.2% (Figure 6D).

#### Stability

To assess sensor stability, variations in ECL intensity were monitored at regular intervals. A slight decrease of 2% in ECL intensity was observed over 10 days, increasing to 7.3% after 20 days. When not in use, the ECL-MIP|DMT electrodes were stored in the dark at room temperatures.

### Mechanistic Understanding from DFT Perspective

**Figure 7:**
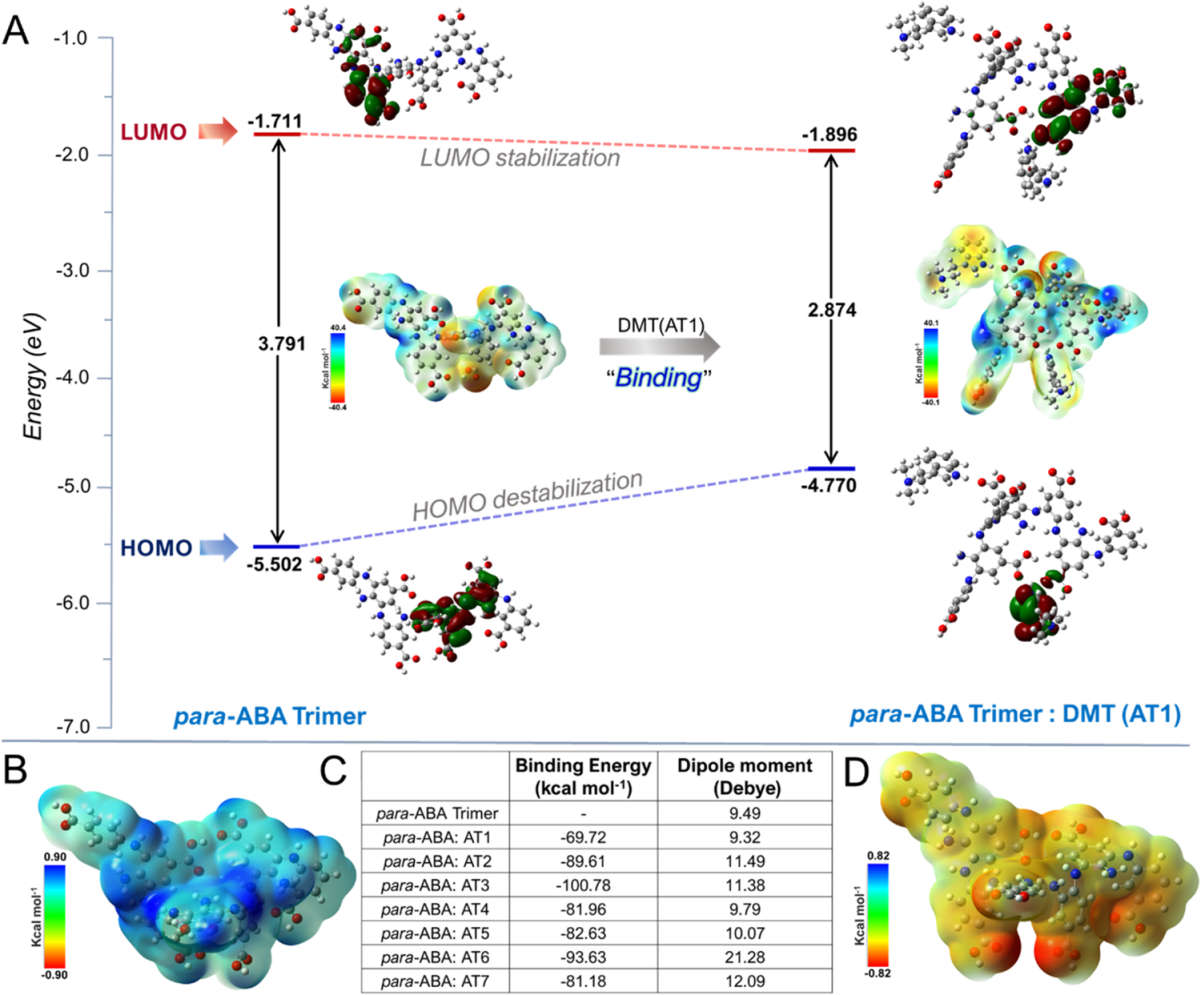
(A) Schematic diagram illustrating the computed energy levels, isodensity (0.02 au) surfaces, and molecular electrostatic potential (ESP) surface plots for the free *p*-ABA trimer and the *p*-ABA/DMT(AT1) combination, obtained from the DFT/B3LYP/6–311G(d, p)/CPCM(CH_3_CN) level of theory. (B) ESP plot of the free *p*-ABA trimer in the oxidized state (−1e^–^). (C) Binding energy and dipole moment of the *p*-ABA trimer with different analytes (ATs). (D) ESP plot of the free *p*-ABA trimer in the reduced state (+1e^*–*^).

To elucidate the proposed mechanism (Scheme 2), DFT calculations were employed to mimic the electropolymerization process using the trimer unit of *p*-ABA, focusing on *ortho*-positional substitution. This substitution induces the significant torsional deviation, with a mean dihedral value of 78°, leading to optimized trimer geometries with inward-facing amino units that are sterically protected and outward-facing carboxylic acid groups with dense electronegative charge density favorable for binding electropositive amino and imino functionalities of the analytes. The trimer unit was specially chosen for these calculations, as polymerization through amination results in an optimized geometry with significant twisting. Before binding, the HOMO electron density is primarily located in the amino-substituted region but shifts to the carboxylic acid sites in the LUMO, confirming intramolecular charge transfer (ICT) in *p*-ABA (Figure 7A) (see Figure S4 in Supporting Information for HOMO, LOMO, and ESP plots of the *p*-ABA/ATs combination).

Further DFT optimization of *p*-ABA at oxidized (cation, −1e^–^) and reduced (anion, *+*1e^–^) states reveal significant electron density distribution compared with the neutral form (Figure S5 in Supporting Information). Molecular electrostatic potential (ESP) analysis indicated an electropositive amino-substituted region and an electronegative carboxylic unit. ESP analysis of DMT and its derivatives (referred to as AT1-AT7, Figure 1) guided the evaluation of binding interactions by positioning the N–H groups of analytes and the carboxylic acid sites of *p*-ABA at a specific distance of 1.65 Å^67−69^ (see Figure S6 in Supporting Information for HOMO, LOMO, and ESP plots for ATs). In this study, two units of analytes were used due to the presence of other heteroatomic functionalities, such as methoxy, keto, ester, and carboxylic acid present at position 5, which can form intermolecular contacts through noncovalent interactions.^67,68^ The binding energy (*E*_b_) of the analytes was calculated using
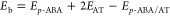
12where *E*_*p-*ABA_ is the energy of the optimized geometry of the *p*-ABA trimer unit, *E*_AT_ is the energy of the optimized geometry of the analytes, and *E*_*p*-ABA:AT_ is the energy of the interaction between the *p*-ABA trimer and analytes. Among the seven analytes studied, the *p*-ABA:DMT (AT1) combination exhibits the lowest binding energy of −69.72 kcal mol^–1^ (Figure 7C), indicating less energy is required for the detachment of AT1 from the *p*-ABA polymer framework. The ESP analysis showed that binding interactions significantly altered the charge distribution of the *p*-ABA π-framework. Increasing the number of heterofunctionalities, such as methoxy, ester, keto, and amino units, significantly enhanced the binding potential (−80 to −100 kcal mol^–1^). After binding with analytes, the HOMO electron density shifted to the analytes, while the LUMO remained in the *p*-ABA framework (see Figure S7 in Supporting Information for HOMO-LOMO shifts from DOS analysis). Figures 7B,D show the ESP plot of the free *p*-ABA trimer in the oxidized (−1e^–^) and reduced (+1e^*–*^) state, respectively (see Figure S8 in Supporting Information for isodensity surface plots of the oxidized state).

Mulliken charge population analysis using a large 6–311++G (d, p) basis set^70^ reveals increased charge density on carbonyl oxygen upon binding, with a significant dipole moment (μ_g_) increase for AT2-AT7 (9.79 to 21.28 D) and a slight decrease for AT1 (9.32 D) as compared to the ground state μ_g_ of *p*-ABA (9.49 D) (Figure 7C) (see Figure S9 in Supporting Information for Mulliken charge density fluctuations). Density of states (DOS) analysis showed that binding with AT1 (i.e., DMT) minimally altered energy levels, supporting weak binding potential and dynamic mobility within the MIP matrix, essential for ECL generation. In contrast, stronger binding in AT2-AT7 hindered interactions with the oxidized ECL emitter. Ultimately, the synergistic combination of DMT’s weak binding potential and lower oxidation potential demonstrates excellent selectivity toward AT1 over the other analytes.

## Conclusions

This work has successfully developed an ECL sensor integrated with a MIP for the selective detection of DMT. Using *p*-ABA as the monomer with DMT as the template molecule, the sensor was fabricated through electropolymerization on a GCE coated with a Nafion-embedded [Ru(bpy)_3_]^2+^ matrix. The resulting sensor exhibited a wide dynamic range for DMT detection (0.5 to 300 μM), a detection limit of approximately 0.5–1.0 μM, and excellent reproducibility, stability, and selectivity. Density functional theory (DFT) analyses indicated that weak binding interactions between DMT and the MIP matrix could contribute to localized vibrational mobility, enhancing selectivity through minimal detachment energy during ECL generation. This work highlights the potential of ECL-MIP technology as a cost-effective and selective approach for detecting specific analytes, particularly those structurally related to the target molecule, and presents opportunities for applications in high-sensitivity detection, including trace analysis of illicit drugs.

## Supporting Information Available

Reagents; electrochemical and ECL setup; fabrication of ECL-MIP⊂DMT sensors; Fourier transfer infrared spectroscopy; electrochemical characterization of GCE|Nafion⊂[Ru(bpy)_3_]^2+^ electrode; AFM image of NIP on FTO and its height profile; computational details; Frontier energy levels such as HOMO, LUMO, isodensity surface, and ESP plots of the *p*-ABA/ATs combination obtained from the ground state optimized geometry; significant torsional deviations endured by the *p*-ABA trimer upon changing the spin states from neutral to charged (+1 or −1e^–^) states; Frontier energy levels such as HOMO, LUMO, isodensity surface, and ESP plots of various analytes (ATs) used in this study; Variation in the HOMO and LUMO shapes and their energetic shifts of the *p*-ABA/ATs combination with reference to the free *p*-ABA trimer obtained from the DOS analysis; isodensity surface plots of the frontier molecular orbitals involved at the oxidized state of the free *p*-ABA trimer optimized geometry; Mulliken charge density fluctuations at the carbonyl oxygen at the *p*-ABA, a responsible site for effective binding with various analytes through secondary noncovalent interactions (PDF)
