# Supplementary material for: Electrogenerated Chemiluminescence Coupled with Molecularly Imprinted Polymer for Sensitive and Selective Detection of N,N-Dimethyltryptamine
Source: Anal Chem. 2025 Mar 14;97(11):6163–74. doi: 10.1021/acs.analchem.4c06886 (PMC11948175; doi:10.1021/acs.analchem.4c06886)
Supplement: Supplementary file 1 — ac4c06886_si_001.pdf [file ac4c06886_si_001.pdf]

Supporting Information

Electrogenerated Chemiluminescence Coupled with Molecularly Imprinted Polymer for Sensitive and Selective Detection of *N*, *N*-Dimethyltryptamine

Jesy Alka Motchaalangaram,[1] Paramasivam Mahalingam,[2,3] Karl J. Wallace,[1] Wujian Miao[1,*]

[1] Department of Chemistry and Biochemistry, [2] School of Polymer Science and Engineering, The University of Southern Mississippi, Hattiesburg, Mississippi 39406, United States; [3] Current address: School of Chemistry and Biochemistry, Georgia Institute of Technology, Atlanta, Georgia 30332, United States; * Corresponding author: Email: wujian.miao@usm.edu; Tel: 601-266 4716

Table of Contents

EXPERIMENTAL SECTION 2
Reagents 2
Electrochemical and ECL Setup 2
Fourier Transform Infrared Spectroscopy (FTIR). 3
Fabrication of ECL-MIP⊂DMT Sensors. 3
RESULTS AND DISCUSSION 4
Fourier Transform Infrared Spectroscopy (FTIR). 4
Electrochemical Characterization of GCE|Nafion⊂$[Ru(bpy)_3]^{2+}$ Electrode. 5
Atomic Force Microscopy (AFM). 6
Computational Details. 6
Figure S4. The frontier energy levels such as HOMO, LUMO, isodensity surface, and ESP plots of the *p*-ABA:ATs combination obtained from the ground state optimized geometry. . 8
Figure S5. The significant torsional deviations endured by the *p*-ABA trimer upon changing the spin states from neutral to charged (+1 or -1e$^-$) states. 9
Figure S6. The frontier energy levels such as HOMO, LUMO, isodensity surface, and ESP plots of various analytes (ATs) used in this study. 10
Figure S7. Variation in the HOMO and LUMO shapes and their energetic shifts of the p-ABA:ATs combination with reference to the free p-ABA trimer obtained from the DOS analysis. 11
Figure S8. The isodensity surface plots of the frontier molecular orbitals involved at the oxidized state of the free p-ABA trimer optimized geometry. 12
Figure S9. Mulliken charge density fluctuations at the carbonyl oxygen at the *p*-ABA, a responsible site for effective binding with various analytes through secondary non-covalent interactions. 13
REFERENCES 14

EXPERIMENTAL SECTION

Reagents. All chemicals used in the study were employed without further purification. Tris-(2,2'-bypyridine)dichlororuthenium(II) hexahydrate ($[Ru(bpy)_3]Cl_2$•$6H_2O$, 99.95%), Nafion solution (~5% mixture of lower aliphatic alcohols and water), 4-aminobenzoic acid (*p*-ABA, ≥ 99%), potassium ferricyanide ($K_3[Fe(CN)_6]$, ≥ 99%, ACS reagent), potassium ferrocyanide ($K_4[Fe(CN)_6]$, ≥ 98%, ACS reagent) were purchased from Sigma-Aldrich. Sodium phosphate monobasic (NaH_2PO_4, 99%) and dibasic ($Na_2H_2PO_4$ 99%) were purchased from J.T. Baker Chemicals Co. Methanol (MeOH, 99.8%, ACS reagent) and acetic acid (HAc, 99.8%) were received from Ficher Scientific and Matheson Coleman & Bell (Baton Rouge, LA), respectively. The analyte *N*, *N*-dimethyl tryptamine (DMT, 1 mg/mL or 5.3 mM in MeOH, DMT purity ≥ 98%) and its derivatives, including 5-methoxy-*N*, *N*-dimethyltryptamine (1 mg/mL in MeOH with ≥ 98% purity), 4-acetoxy-*N*, *N*-dimethyltryptamine•HCl (≥ 98%), 5-acetyl tryptamine (≥ 98 %), tryptamine (≥ 98%), melatonin (≥ 98%), and indole-5-carboxylic acid (≥ 98%) were sourced from Cayman Chemical Company. Phosphate buffer solution (PBS, 0.10 M, pH = 7.4) was prepared using NaH_2PO_4 and Na_2HPO_4, with the pH adjusted using 0.10 M NaOH and 0.10 M H_3PO_4. For chemicals initially dissolved in MeOH, the solvent was evaporated slowly under a stream of N_2 gas before they quantitively re-dissolved in PBS (pH 7.4) for further use. This step was necessary to prevent MeOH from oxidizing during the positive potential scans, which could interfere with the electrochemical studies of compounds of interest.

Electrochemical and ECL Setup. Electrochemical measurements were conducted using a CH Instruments Model 660A electrochemical workstation with a three-electrode system. This system comprised a bare or surface modified glassy carbon electrode (GCE ~3 mm diameter) as the working electrode, an Ag/AgCl (3.0 M KCl) reference electrode, and a platinum mesh counter

electrode. The solution was degassed for 5 mins with high-purity nitrogen gas (supplied by Airgas) to minimize background signals during potential cycling in the negative direction. Cyclic voltammetry (CV) and ECL measurements were recorded instantaneously using a homemade CV-ECL electrochemical setup, where the Model 660A CH electrochemical workstation was coupled with a Hamamatsu R928 photomultiplier tube, biased at -700 V DC and housed in a black light-tight box.

Fourier Transform Infrared Spectroscopy (FTIR). FTIR was used to identify the functional groups of the *p*-ABA monomer and its polymer counterpart. Measurements were performed using a Thermo Fisher Everest™ Nicolet™ Summit diamond crystal ATR-FTIR spectrometer, scanning across the 400 to 4000 cm^{-1} range with 32 scans. The polymer containing the analyte DMT (i.e., MIP⊂DMT, as described below) was electrodeposited onto a fluorine-doped tin-oxide (FTO) coated glass substrate (MTI Corp.) from a solution containing 4.0 mM *p*-ABA, 1.0 mM DMT, and 0.10 M PBS (pH 7.4). The deposition was carried out by cycling the potential from 0 to 1.5 V vs Ag/AgCl for 10 cycles.

Fabrication of ECL-MIP⊂DMT Sensors. Scheme 1 in the manuscript illustrates the fabrication of the ECL-MIP⊂DMT sensor. Experimentally, a 20 µL aliquot of Nafion solution was mixed with 2.0 mL of 0.5 mM $[Ru(bpy)_3]Cl_2$ in a 50/50 (v/v) methanol/H_2O solution, followed by sonication. Approximately 7 µL of this solution was then drop-cast onto a 3-mm diameter glassy carbon electrode (GCE) surface and allowed to air dry for ~30 min before use. The resulting electrode is designated as GCE|Nafion⊂$[Ru(bpy)_3]^{2+}$, where '|' denotes the interface between the GCE and the Nafion film, and '⊂' indicates the inclusion of $[Ru(bpy)_3]^{2+}$ within the Nafion matrix (Scheme 1A). The MIP films were then developed by immersing the GCE|Nafion⊂$[Ru(bpy)_3]^{2+}$ electrode into a solution containing 4.0 mM *p*-ABA and 1.0 mM DMT in 0.10 M PBS (pH 7.4)

for electropolymerization. The fully loaded electrode with DMT molecules is designated as GCE|Nafion⊂$[Ru(bpy)_3]^{2+}$|$(p\text{-ABA})_n$⊂$DMT_{saturated}$, simplified as ECL-MIP⊂DMT_{satd} (Scheme 1B). To form DMT-specific binding nanocavities within the MIP film, the electrode was dipped into a 5.0 mL of MeOH/HAc mixture solution (3/1, v/v) with gentle stirring for ~5 mins to remove the embedded DMT molecules. Afterwards, the electrode was rinsed with PBS buffer and air-dried, yielding the ECL-MIP⊂DMT_{cavity} electrode (Scheme 1C). In the presence of the target analyte DMT, some of the nanocavities rebind with DMT, and the concentration of DMT can be sensitively detected through ECL (ECL-MIP⊂DMT, Scheme 1D).

RESULTS AND DISCUSSION

Fourier Transform Infrared Spectroscopy (FTIR). The carboxylic group and the primary amine moiety are two excellent functionalities to help characterize the successful electropolymerization of *p*-ABA. The FTIR spectra obtained for the *p*-ABA monomer (Figure S1(a)) closely matched previously reported data,[1] whereby, two sharp stretching vibrational modes of the primary amino (NH_2) are observed at ~3459 cm^{-1} and ~3360 cm^{-1}. This suggests that the amino group is found in its "free" state, i.e., is not involved in extensive hydrogen bonding in the solid state. Whereas the carboxylic group is extensively involved in hydrogen bonding.

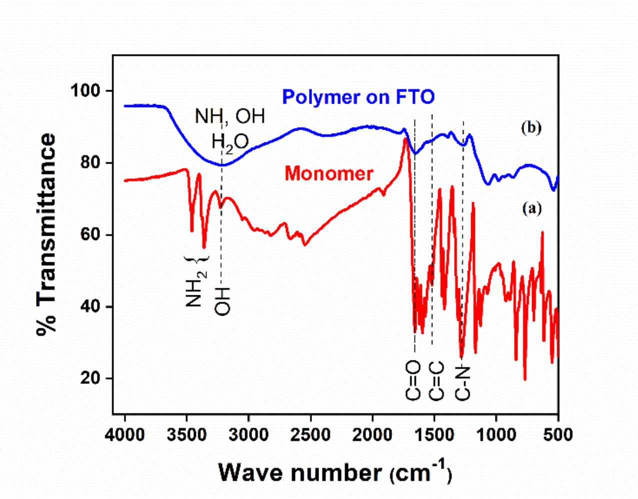

Figure S1. ATR-FTIR spectra of (a) *p*-ABA monomer and (b) *p*-ABA polymer electrodeposited on a FTO substrate.

The weak band at 3227 cm^{-1} is the stretching frequency of the OH group found in its "free" state and the very broad stretching region around 3000-2800 cm^{-1}, is attributed to the O-H group,

involved in hydrogen bonding, either as dimers or in the polymeric form. The strong C=O stretching of carboxylic acid appears at ~1658 cm^{-1},[2] shifted to lower wavenumbers by 50 units, supporting the existence of hydrogen bonding interactions in the solid state. Other vibrational bands see between 1590 and 1540 cm^{-1} are attributed to the C=C stretching of the benzene ring in *p*-ABA, and the C-N bond appears at ~1285 cm^{-1}. In contrast to the FTIR spectra of the monomer, the polymer spectrum, shown in Figure S1(b), displays a broad band centered around ~3215 cm^{-1}, indicating the transformation of the amino functionalities in *p*-ABA from primary to secondary during electropolymerization[3-5] (see **Electropolymerization of *p*-ABA** section in main text for further explanation).

Electrochemical Characterization of GCE|Nafion⊂$[Ru(bpy)_3]^{2+}$ Electrode. The GCE|Nafion⊂$[Ru(bpy)_3]^{2+}$ electrode was characterized using CV technique in 0.10 M PBS (pH 7.4) at varying scan rates. As shown in Figure S2, the $[Ru(bpy)_3]^{2+}$ complex which is trapped within the Nafion film shows a typical solution-phase reversible redox reaction, with anodic and cathodic peak potentials observed at ~1.11 V and ~1.04 V vs Ag/AgCl, respectively, at a low scan rate of 10 mV/s. As the scan rate increases from 10 to 150 mV/s, the peak separation (ΔE_p) broadens from ~70 to ~120 mV due to the *iR* drop. Additionally, both anodic and cathodic peak currents show a linear relationship with the square root of scan rate (Insert in Figure

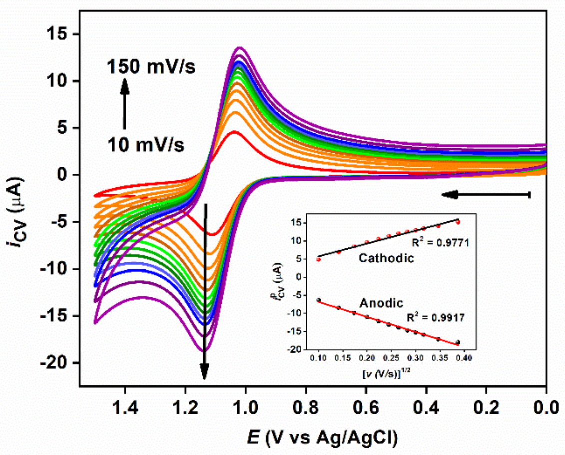

Figure S2. Cyclic voltammograms of GCE|Nafion⊂$[Ru(bpy)_3]^{2+}$ electrode placed in 0.10 M PBS (pH 7.4) at various scan rates. Insert: Linear relationship between the anodic and cathodic peak current and the square root of scan rate.

S2), confirming that the $[Ru(bpy)_3]^{2+}$ ions confined within the Nafion film follow a diffusion-controlled redox process.[6-8]

Atomic Force Microscopy (AFM). FTO plates were initially cleaned with a soap solution and subsequently ultrasonicated in a 1:1 ethanol/distilled water mixture for 15 mins. For AFM measurements, the FTO substrates were electropolymerized in a solution containing 4.0 mM p-ABA, 1.0 mM DMT, and 0.10 M PBS (pH 7.4). Electropolymerization was performed over a potential range of 0 to 1.5 V vs Ag/AgCl for 10 cycles. AFM measurements were carried out using an NTEGRA Prima AFM from NT-MDT. Figure S3 shows (A) an AFM image and (b) the height profile of a non-imprinted polymer (NIP) modified FTO electrode.

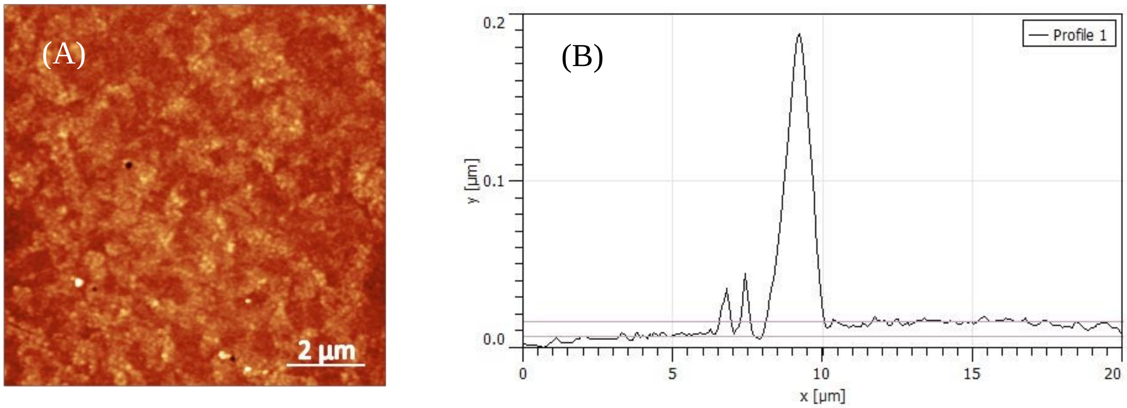

Figure S3. (A) AFM image and (B) height profile of an NIP modified FTO electrode.

Computational Details. All calculations in this study were conducted using the framework of density functional theory (DFT) to qualitatively evaluate the binding interactions between the *p*-ABA polymer and various analytes, using the Gaussian 16 ab initio software package.[9] The trimer unit of the *p*-ABA polymer was selected for these calculations, as polymerization through amination results in an optimized geometry with significant twisting.

Ground-state optimization of the *p*-ABA trimer and analytes was employed in both gas and solution phases at the DFT/B3LYP/6-311G (d, p) level of theory, without imposing any symmetrical constraints. The optimized geometries obtained were subjected to vibrational analysis to confirm stability via global minima on the potential energy surface, indicated by the absence of imaginary frequencies. These structural coordinates were further used as inputs for single-point calculations to generate isodensity surface, electrostatic potential (ESP) surface plots, Mulliken population analysis, and assessments of binding interactions between the *p*-ABA and analytes.[10]

Density of states (DOS) calculations were performed to examine shape variations and energy shifts in the HOMO and LUMO orbitals of the *p*-ABA : analyte complexes, compared to the free *p*-ABA trimer unit.[11, 12] DOS simulations were executed using GaussSum 3.0.2 software. Binding interactions between the *p*-ABA trimer and analytes were modeled through a specific arrangement of O···H-N bonds, maintaining a minimum energy distance of 1.65 Å, based on experimental parameters and DFT predictions.[13-15]

Grimme’s dispersion correction (GD3) was applied with Becke-Johnson dumping scheme to yield accurate secondary non-covalent interactions. Besides *imino*-nitrogen of indole, there are other reactive sites possessing heteroatomic functionalities which possibly can bind with the amino and acidic sites of the *p*-ABA trimer.

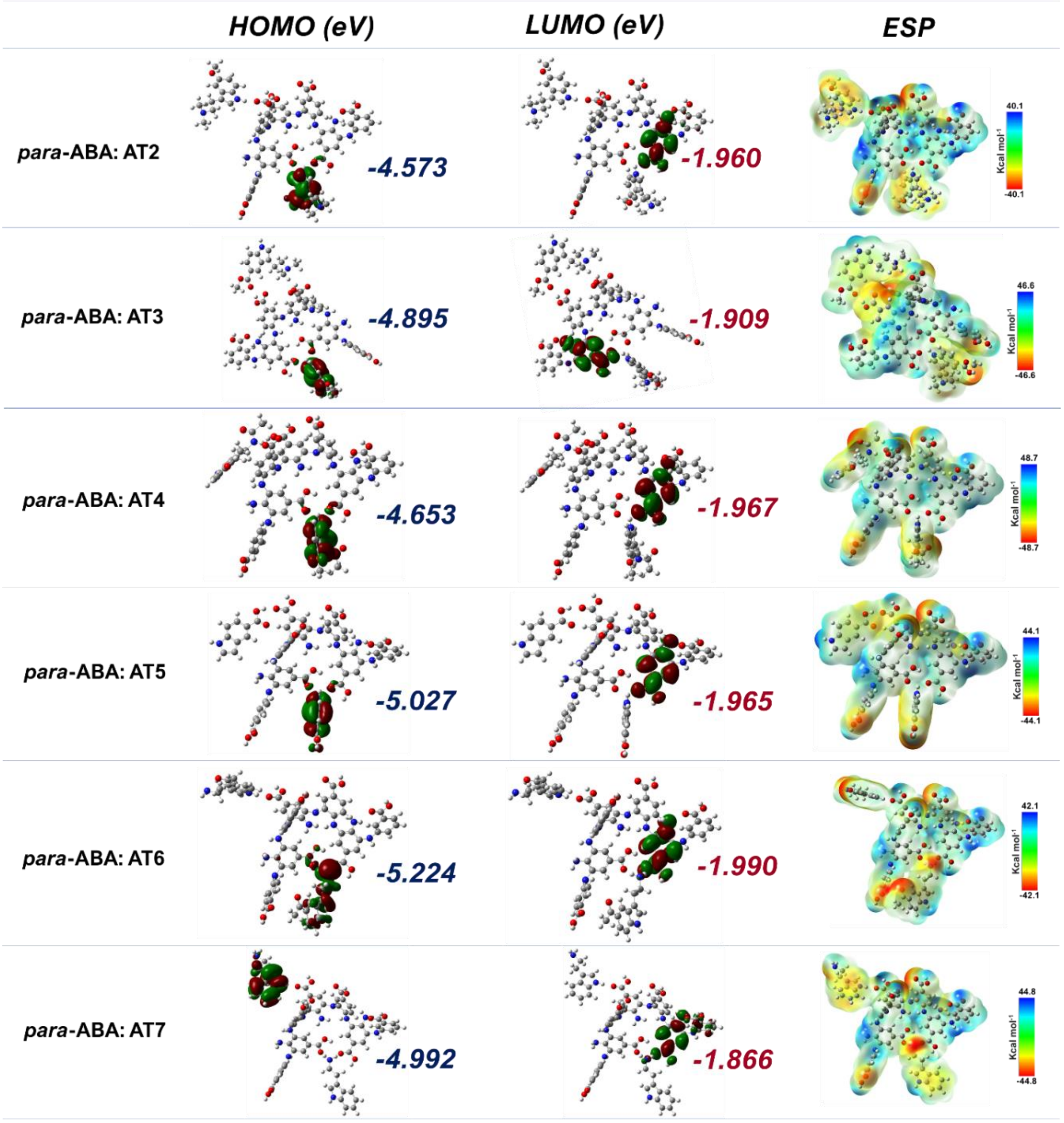

Figure S4. The frontier energy levels such as HOMO, LUMO, isodensity surface, and ESP plots of the *p*-ABA:ATs combination obtained from the ground state optimized geometry.

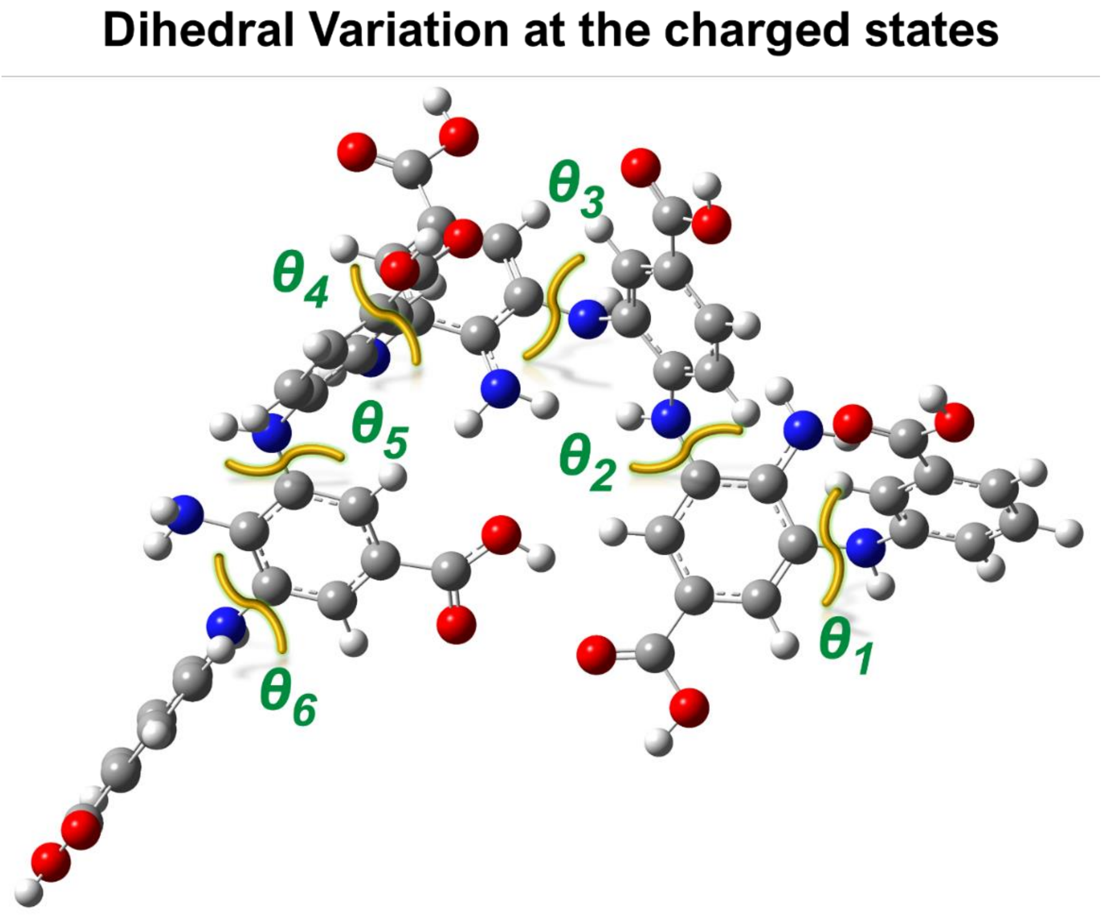

	Neutral	Cation (Oxidized state)	Anion (Reduced state)
θ_1	77.23	74.72	81.02
θ_2	76.63	77.32	78.69
θ_3	37.67	42.78	38.64
θ_4	98.62	51.53	89.99
θ_5	73.78	67.31	70.99
θ_6	86.40	73.47	73.66

Figure S5. The significant torsional deviations endured by the *p*-ABA trimer upon changing the spin states from neutral to charged (+1 or $-1e^-$) states.

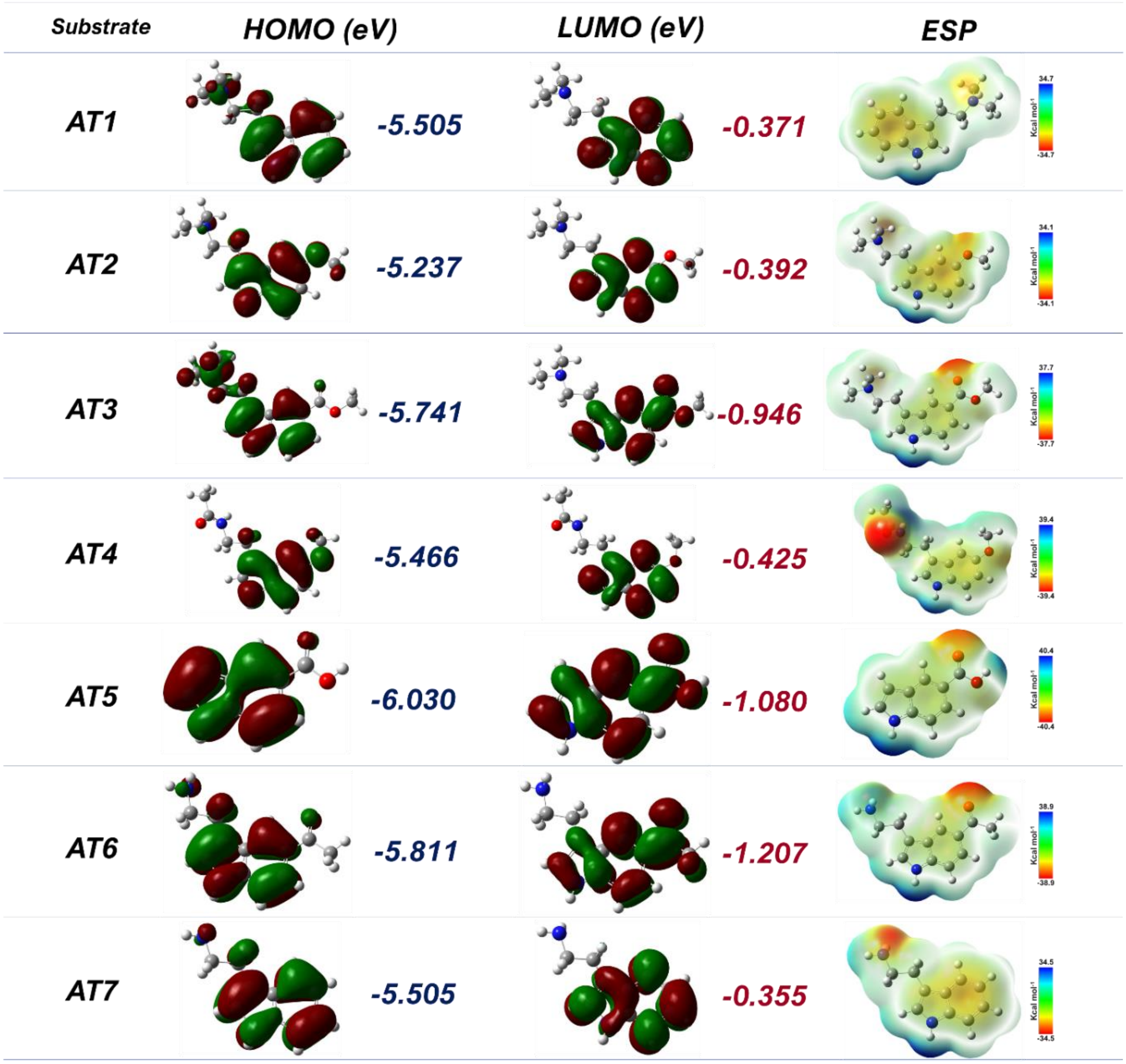

Figure S6. The frontier energy levels such as HOMO, LUMO, isodensity surface, and ESP plots of various analytes (ATs) used in this study.

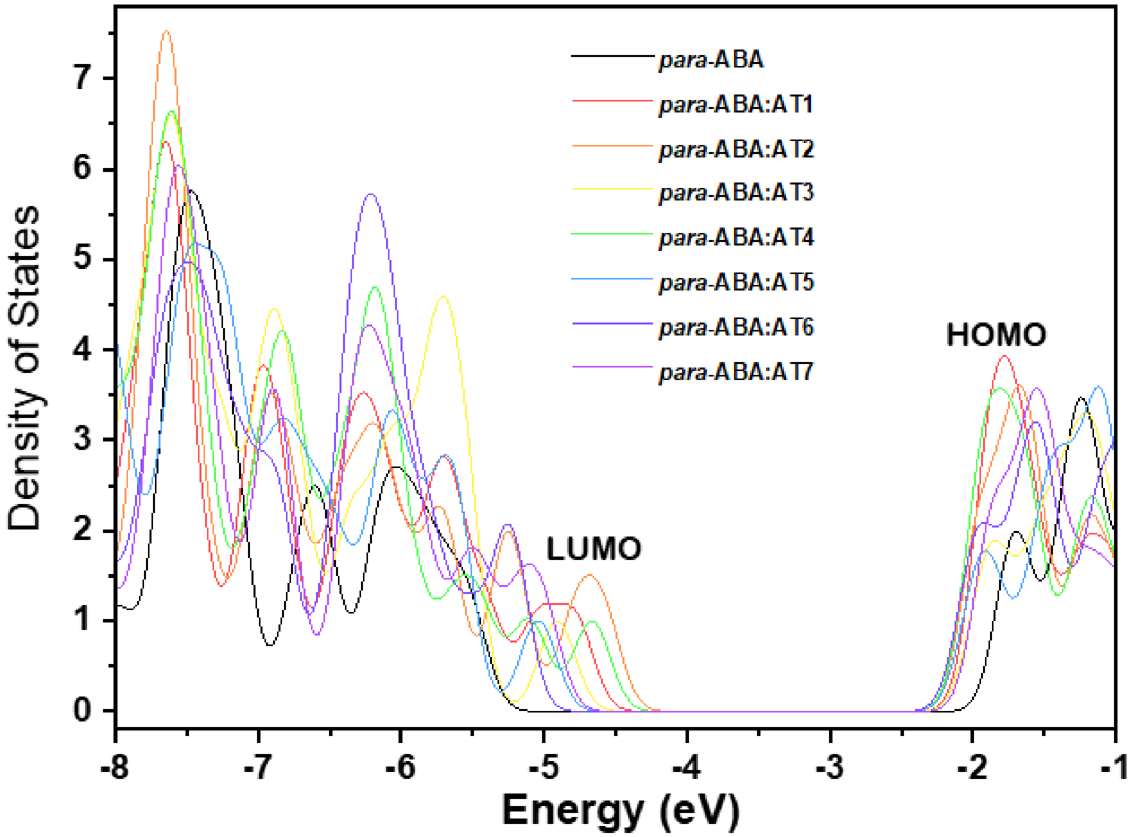

Figure S7. Variation in the HOMO and LUMO shapes and their energetic shifts of the p-ABA:ATs combination with reference to the free p-ABA trimer obtained from the DOS analysis.

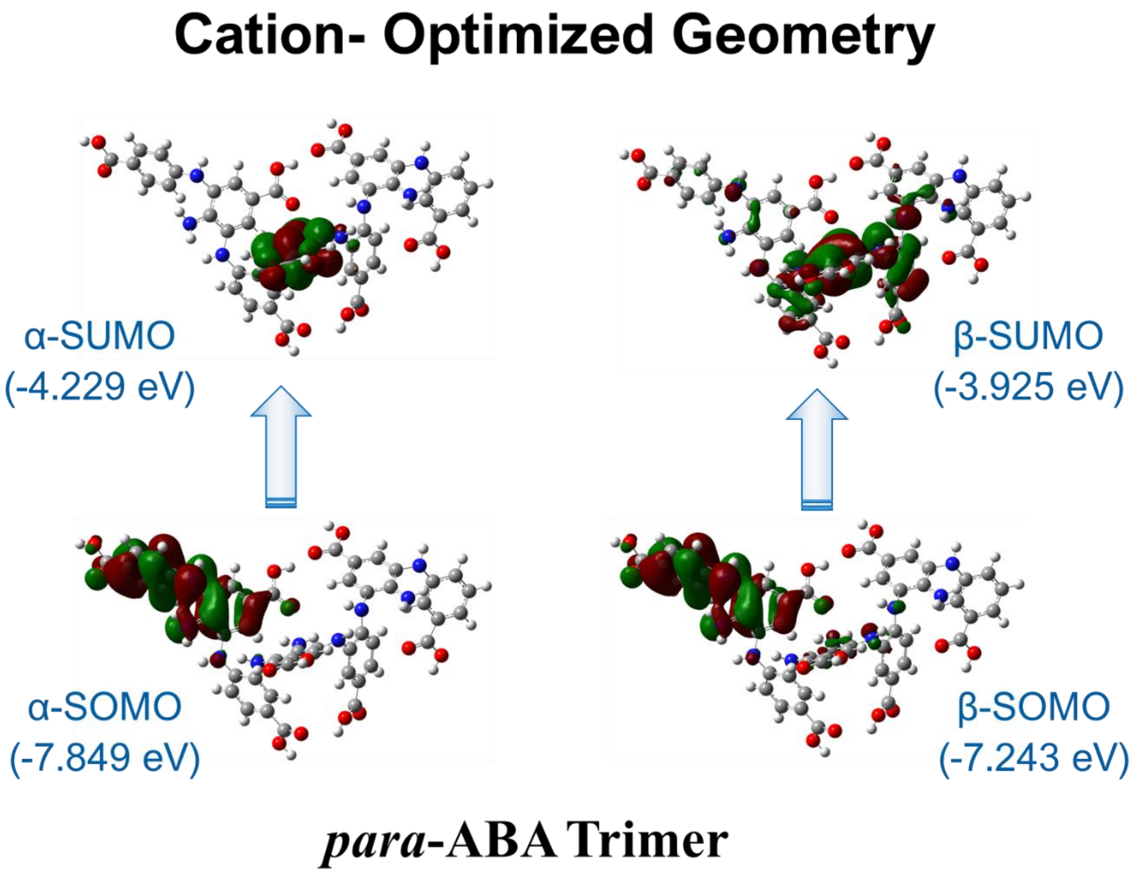

Figure S8. The isodensity surface plots of the frontier molecular orbitals involved at the oxidized state of the free p-ABA trimer optimized geometry.

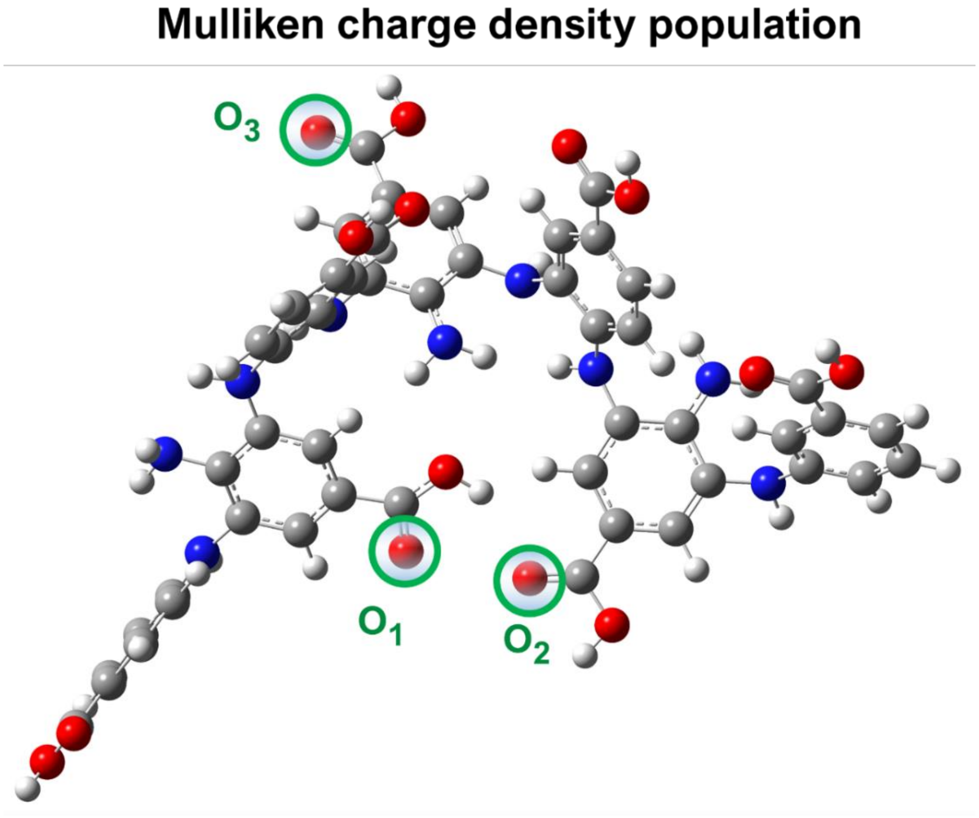

	O_1	O_2	O_3
para-ABA Trimer	-0.365	-0.347	-0.348
para-ABA: AT1	-0.381	-0.410	-0.397
para-ABA: AT2	-0.405	-0.379	-0.396
para-ABA: AT3	-0.409	-0.383	-0.373
para-ABA: AT4	-0.410	-0.381	-0.414
para-ABA: AT5	-0.411	-0.383	-0.412
para-ABA: AT6	-0.389	-0.378	-0.396
para-ABA: AT7	-0.388	-0.385	-0.403

Figure S9. Mulliken charge density fluctuations at the carbonyl oxygen at the *p*-ABA, a responsible site for effective binding with various analytes through secondary non-covalent interactions.

REFERENCES

(1) NIST Chemistry WebBook-Standard Reference Database 69. U.S. Department of Commerce. https://webbook.nist.gov/chemistry/ (accessed 1/6/2025).

(2) Ghosh, D.; Luwang, M. N. p-Aminobenzoic Acid (pABA) Sensitization of $LaF_3:Tb^{3+}$ Nanoparticles and Its Applications in the Detection of Explosive Materials. *RSC Adv.* **2015**, *5* (14), 10468-10478. DOI: 10.1039/C4RA15304A.

(3) Santos, C. d. C.; Pimenta, T. C.; Thomasini, R. L.; Verly, R. M.; Franco, D. L.; Ferreira, L. F. Electropolymerization of Phenol and Aniline Derivatives: Synthesis, Characterization and Application as Electrochemical Transducers. *J. Electroanal. Chem.* **2019**, *846*, 113163. DOI: 10.1016/j.jelechem.2019.05.045.

(4) Ganjeizadeh Rohani, F.; Mohadesi, A.; Ansari, M. Electrochemical Synthesis, Characterization, and Spectroelectrochemical Evaluation of Poly(para Amino Benzoic Acid-Co-4,4-Diaminodiphenyl Sulfone) Film. *J. Mater. Sci.* **2019**, *30* (9), 8686-8697. DOI: 10.1007/s10854-019-01192-5.

(5) Sayyah, S. M.; Azooz, R. E.; Abd El-Rehim, S. S.; El-Rabiey, M. M. Electropolymerization of o-Aminobenzoic Acid and Characterization of the Obtained Polymer Films. *Int. J. Polym. Mater. Polym. Biomater.* **2006**, *55* (1), 37-63. DOI: 10.1080/009140390909763.

(6) Lin, R.-J.; Onikubo, T.; Nagai, K.; Kaneko, M. Investigation of $Ru(bpy)_3^{2+}$/Nafion® Film Coated on Electrodes Studied Using In Situ Spectrocyclic Voltammetry and Photoluminescence. *J. Electroanal. Chem.* **1993**, *348* (1), 189-199. DOI: 10.1016/0022-0728(93)80131-Z.

(7) Gálvez-Vázquez, M. J.; Gárate-Morales, J. L.; Cerna-Cortez, J. R.; Aguilar-Sánchez, R. Electrochemistry of $[Ru(bpy)_3]^{2+}$ and $[Ru(phen)_3]^{2+}$ Inserted in Nafion Membranes Studied in the Ionic Liquid $HMImPF_6$. *Int. J. Electrochem. Sci.* **2020**, *15*, 12548-12558. DOI: 10.20964/2020.12.78.

(8) Martin, C. R.; Rubinstein, I.; Bard, A. J. Polymer Films on Electrodes. 9. Electron and Mass Transfer in Nafion Films Containing Tris(2,2'-bipyridine)Ruthenium(2+). *J. Am. Chem. Soc.* **1982**, *104* (18), 4817-4824. DOI: 10.1021/ja00382a014.

(9) Gaussian 16. Expanding the Limits of Computational Chemistry. **2019, July 23;** https://gaussian.com/citation/.

(10) Paramasivam, M.; Chitumalla, R. K.; Singh, S. P.; Islam, A.; Han, L.; Jayathirtha Rao, V.; Bhanuprakash, K. Tuning the Photovoltaic Performance of Benzocarbazole-Based Sensitizers for Dye-Sensitized Solar Cells: A Joint Experimental and Theoretical Study of the Influence of π-Spacers. *J. Phys. Chem. C* **2015**, *119* (30), 17053-17064. DOI: 10.1021/acs.jpcc.5b04629.

(11) Paramasivam, M.; Chitumalla, R. K.; Jang, J.; Youk, J. H. The Impact of Heteroatom Substitution on Cross-Conjugation and Its Effect on the Photovoltaic Performance of Dsscs – A Computational Investigation of Linear vs. Cross-Conjugated Anchoring Units. *Phys. Chem. Chem. Phys.* **2018**, *20* (35), 22660-22673. DOI: 10.1039/C8CP02709A.

(12) Mahalingavelar, P. How End-Capped Acceptors Regulate the Photovoltaic Performance of the Organic Solar Cells: A Detailed Density Functional Exploration of Their Impact on The A–D−π–D–A Type Small Molecular Electron Donors. *Energy Fuels* **2022**, *36* (4), 2095-2107. DOI: 10.1021/acs.energyfuels.1c04272.

(13) Grabowski, S. J. Theoretical Studies of Strong Hydrogen Bonds. *Annu. Rep. Prog. Chem., Sect. C: Phys. Chem.* **2006**, *102*, 131-165, 10.1039/B417200K. DOI: 10.1039/B417200K.

(14) Desiraju, G. R. Hydrogen Bonds and Other Intermolecular Interactions in Organometallic Crystals. *Dalton Trans.* **2000**, (21), 3745-3751, 10.1039/B003285I. DOI: 10.1039/B003285I.

(15) Desiraju, G. R.; Steiner, T. *The Weak Hydrogen Bond In Structural Chemistry and Biology*; Oxford University Press, 2001. DOI: 10.1093/acprof:oso/9780198509707.001.0001.
